# Improvement of Electrospray Ionization Response Linearity and Quantification in Dissolved Organic Matter Using Synthetic Deuterated Internal Standards

**DOI:** 10.1021/acs.analchem.5c02463

**Published:** 2025-08-22

**Authors:** Alexander J. Craig, Mustapha A. Ganiyu, Lindon W. K. Moodie, Sofja Tshepelevitsh, Koit Herodes, Heike Simon, Thorsten Dittmar, Jeffrey A. Hawkes

**Affiliations:** † Analytical Chemistry, Department of Chemistry BMC, 8097 Uppsala University, Uppsala 75 237, Sweden; ‡ Drug Design and Discovery, Department of Medicinal Chemistry, Uppsala University, Uppsala 752 37, Sweden; § Institute of Chemistry, 37546 University of Tartu, Ravila 14a, 50411 Tartu, Estonia; ∥ Marine Geochemistry, Institute for Chemistry and Biology of the Marine Environment (ICBM), Carl von Ossietzky Universität Oldenburg, Oldenburg 26129, Germany; ⊥ Helmholtz Institute for Functional Marine Biodiversity (HIFMB) at the Carl von Ossietzky Universität Oldenburg, 26129 Oldenburg, Germany

## Abstract

Aquatic dissolved organic matter (DOM) is an ultracomplex mixture of compounds covering a wide range of masses and with an unknown extent of isomeric complexity, making its structural elucidation and quantification highly challenging. Electrospray ionization high-resolution mass spectrometry (ESI-HRMS) has advanced DOM analysis, but accurate concentration determination remains limited by the lack of a response factor correction. Here, we address this limitation by introducing novel deuterated compounds as internal standards that mimic DOM structures. Using a *d*_5_-labeled compound free of isobaric interferences in DOM, we assessed ionization suppression in various aquatic sample extracts and improved concentration-based linearity in a coastal DOM reference material. Our results show that deuterated carboxylic acid-rich standards enable “pseudoquantification” by correcting for ionization suppression and instrument drift. Applying this approach, we estimate that DOM consists of 20–30% acids in river, coastal, and deep-ocean reference samples using an Orbitrap system. The same samples were estimated to contain 11–24% acids using 15T FT-ICR-MS, highlighting platform differences. Additionally, we establish a ∼1 ng L^–1^ feature detection limit for DOM compounds in seawater via a standard LC-MS gradient method. These findings demonstrate that deuterated standards provide a simple, practical way to improve DOM pseudoquantification, enhancing our understanding of its chemical composition in environmental studies.

## Graphical abstract

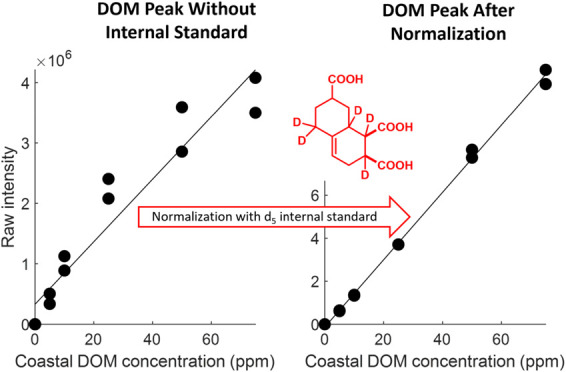

## Introduction

Molecular analysis of aquatic environmental samples is highly complicated due to the ultradiverse nature of the mixtures of organic compounds that make up ‘dissolved organic matter’ (DOM). This sample complexity leads to interpretation challenges for structural elucidation,^1^ sample characterization,^2^ and also any type of targeted^3^ or nontargeted^4^ quantification. Complex mixture analysis is an active and highly challenging area of research,^5^ testing the limitations of cutting-edge analytical techniques such as high-resolution mass spectrometry (HRMS). HRMS has made incredible advances in recent years in terms of instrument availability,^6^ coupled separation technique development,^7–10^ and mass resolving power.^11–13^ However, a critical problem remains in accounting for differences in the ‘response factor’, which can be used to convert the signal intensity into the analyte concentration, combining ionization efficiency^14,15^ and ion transfer efficiency prior to detection. Moreover, ion signals generated from complex DOM samples are mixtures of isomers with unknown diversity,^16–18^ making it extremely difficult to translate the analytical signals gained from HRMS into individual compound concentrations.

DOM is present at a minimum of 34 μM carbon throughout the world’s oceans,^19^ with concentrations increasing in surface waters to exceed 80 μM carbon in some regions. The total pool of carbon is usually operationally divided into three major reactivity fractions: labile, semilabile, and recalcitrant DOM, where the semilabile pool is high in surface waters, and the recalcitrant pool is close to uniform concentrations throughout.^19,20^ Understanding the nature, source, and chemical stability of the recalcitrant pool of DOM has been one of the key research topics in biogeochemistry for decades,^19–24^ but its research is severely hampered by current analytical limitations.

The recalcitrant and semilabile DOM pools are both composed of an unknown number of individual molecules of unknown concentrations. The actual amount of any individual compound is almost impossible to determine, as the mixture is unresolvable by current analytical techniques,^4,7,16,17,25^ and commercial standards do not exist for even a minute fraction of them.^26^ Ultrahigh-resolution mass spectrometry is able to resolve the mixture into tens of thousands of molecular formulas,^13^ and coupling chromatography^17^ or ion mobility^16^ to mass spectrometry allows insight into the isomeric diversity of each formula, but the isomers cannot be fully separated for the vast majority of DOM formulas. It therefore follows that the actual number of structures in marine DOM is at an absolute minimum of 10^5^, but probably exceeds 10^6^ or 10^7^. Such diversity is easy to achieve theoretically based on structural isomerism,^2^ including regio- and stereoisomerism,^26^ but the source of such complexity is poorly understood.

The majority of research in this field uses a sample preparation and analysis pipeline that is biased toward the analysis of acids, namely, solid-phase extraction (SPE) at pH 2 on a hydrophobic resin, followed by electrospray ionization (ESI) in negative mode. This method is useful because it is highly robust and sensitive and generates the above-mentioned signal diversity, which is ideal for compositional analysis and multivariate statistics. Nuclear magnetic resonance (NMR) spectroscopy shows that the carbon functionality in SPE marine DOM is dominated by aliphatic structures, carboxylic acid groups, and sp^3^ oxygen functionalities, and tandem mass spectrometry has shown that the ions were generated in the negative-mode ESI fragment with multiple losses of 44 and 18 (CO_2_ and H_2_O) from acid groups at all tested mass ranges.^18,27,28^ It is, however, important to acknowledge that the sample coverage is by no means complete and focuses attention on acid-containing DOM.

Here, we aimed to improve our understanding of the analytical signals gained by negative-mode ESI-HRMS in a variety of ways by using novel deuterated internal standards synthesized for this purpose. Specifically, we aimed to use modern HRMS techniques to determine

1. whether ion peak intensities vary linearly with the concentration;
2. the extent of ionization suppression in complex mixtures;
3. whether multiacids in the mass range of DOM have predictable response factors; and
4. the apparent detection limit and apparent concentration of carboxylic acids within complex DOM samples.

Our overall research aim is to assess the possibility of adding quantitative information to the highly rich qualitative information gained by HRMS, even if only to highlight the limitations and pitfalls of this type of approach. We find that adding compositionally relevant internal standards to HRMS analysis greatly improves linearity and allows more accurate “pseudoquantification” of peaks in DOM.

## Methods

### Synthesis

Racemic *d*_*2*_-, *d*_*3*_-, and *d*_*5*_-labeled equivalents of *d*_*0*_-**1** (Figure 1A) were synthesized (*d*_*2*_-**1**, *d*_*3*_-**1**, and *d*_*5*_-**1**), with *d*_*0*_-**1** having been previously prepared (listed in our previous work^26^ as compound 13). *d*_*5*_-**1** as a diastereomeric mixture (referred to here as *d*_*5*_-**1mix**) was subjected to preparative high-pressure liquid chromatography (HPLC) to isolate single isomers. From this, two single peaks (based on LC-MS data) were obtained, with the early eluting peak being denoted as *d*_*5*_-**1a** and the later eluting peak being denoted as *d*_*5*_ **-1b**. While ^1^H NMR confirmed that *d*_*5*_-**1a** and *d*_*5*_-**1b** were individual diastereomers, the specific stereochemistry of these compounds could not be unambiguously determined as the high deuterium content in the molecule limited the information available from nuclear Overhauser effect spectroscopy and coupling constant analysis. Furthermore, the low amounts of material recovered (*d*_*5*_-**1a**: 2.2 mg, *d*_*5*_-**1b**: 1.3 mg) led to us being unable to generate sufficient signal-to-noise to provide ^13^C NMR spectra for these individual isomers. For *d*_*5*_-**1a**, ^13^C NMR data has been inferred based on data from *d*_5_-**1mix**, where it is present as the major diastereomer as confirmed by ^1^H and ^13^C NMR spectroscopy, as well as through inferring several of its ^13^C signals through the use of 2D spectroscopies.

**Figure 1:**
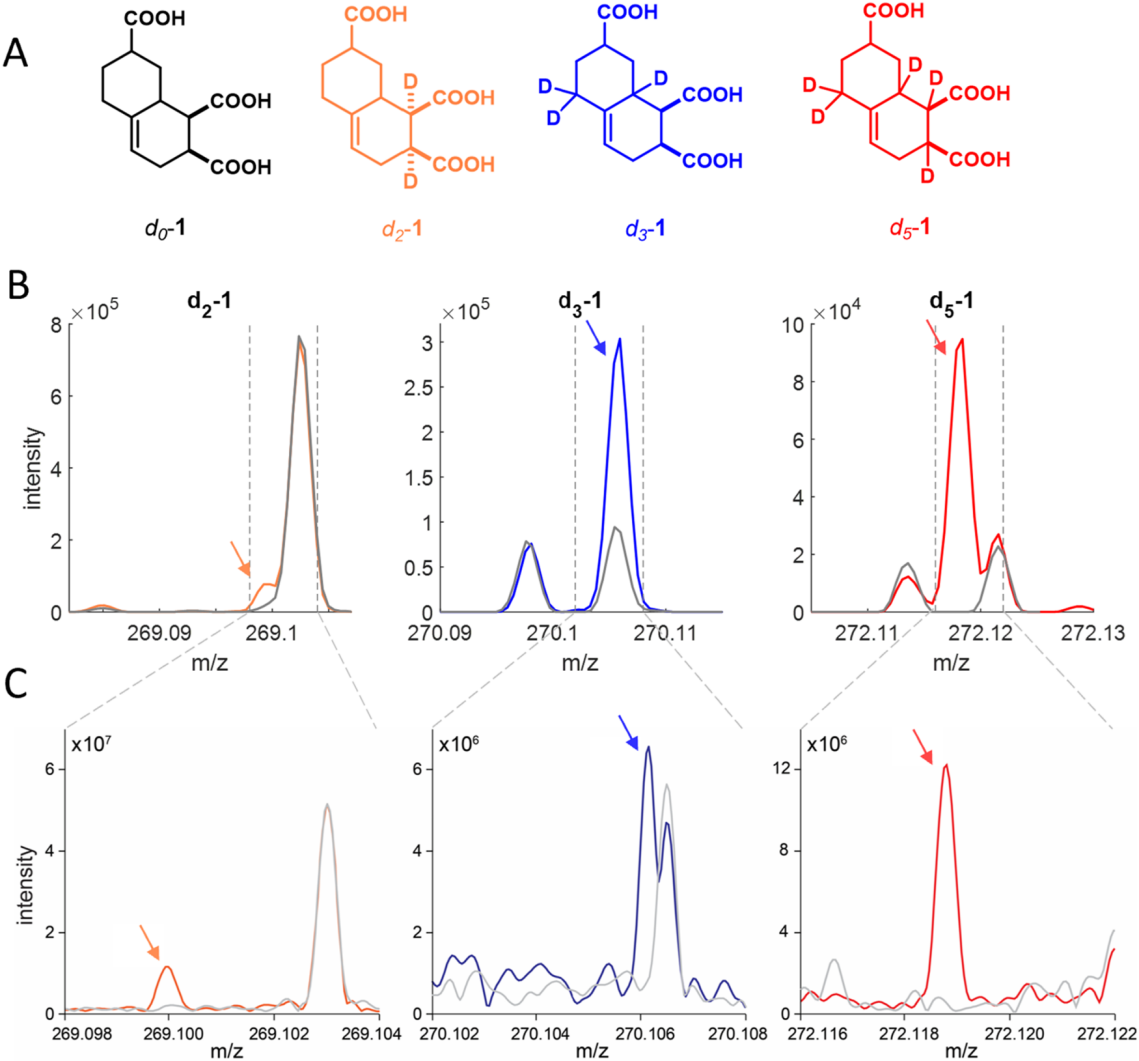
(A) Parent molecule *d_0_*-**1** and the corresponding deuterated carboxylate-rich alicyclic molecule (CRAM) standards *d_2_*-**1**, *d_3_*-**1**, and *d_5_*-**1mix**. (B) Mass spectra over 25 mDa at the retention time corresponding to internal standard elution without and with spiked *d_2_*-**1**, *d_3_*-**1**, and *d_5_*-**1mix** at 1:10000 addition (on a mass basis, indicated with an arrow and by a colored spectrum) to a complex coastal sample (TRM; gray), using QE Orbitrap MS in an LC-MS analysis. (C) Mass spectra over 6 mDa in direct infusion mode without and with spiked *d_2_*-, *d_3_*-, and *d_5_*-**1mix** at 1:5000 addition (on a mass basis, indicated with an arrow and by a colored spectrum) to a complex coastal sample (TRM; gray), using 15T FT-ICR-MS. In panels (B) and (C), note the differing response range extents between samples and platforms.

All synthetic and purification procedures as well as associated spectra are provided in the Supporting Information (including for all intermediates).

### Flow Injection Data

Suwannee River Fulvic Acid (SRFA; herein, river sample (1)), Suwannee River Natural Organic Matter (SRNOM; herein, river sample (2)), Tjärnö Reference Material (TRM-0522; herein, coastal sample),^29^ and North Equatorial Pacific Intermediate Water (NEqPIW; herein, deep-seawater sample)^30^ DOM reference materials were all obtained dried into vials or as powders. Subsequently, they were prepared to 1 mg mL^–1^ in 50:50 methanol/H_2_O (LC-MS grade), before being diluted to 50 mg L^–1^ in 50:50 methanol/H_2_O for direct injection analysis with *d*_*5*_-**1a** added as part of the dilution to make solutions for the calibrations between 0.5 and 100 ppb. Calibration series were investigated for all four matrices (the four DOM sources and pure solvent) at 0.5, 1, 1.5, and 2 ppb and 5, 10, 15, and 20 ppb in a 50 ppm DOM matrix. Additionally, *d*_*5*_-**1b** and *d*_*5*_-**1mix** were analyzed in duplicate over the 5–20 ppb range for comparison with *d*_*5*_-**1a**, and, finally, *d*_*5*_-**1a** was kept constant at 10 ppb, while the concentration of coastal DOM was varied from 5 to 75 ppm to test for the linearity of other DOM peaks when a compound like *d*_*5*_-**1a** is used as an internal standard.

Analysis was conducted using a UPLC system (Vanquish, Thermo Fisher) coupled to a QE Orbitrap mass spectrometer (Thermo Fisher). The ESI (Ion Max API HESI) source was operated in negative mode at 3.5 kV, 200 °C heater temperature, sheath flow 20, and auxiliary gas 2 (both arbitrary units). The capillary temperature was set to 300 °C and S-lens was set to 50 (arbitrary). The automatic gain control target was set to 3 × 10^6^ with a maximum trapping time of 200 ms at an *m*/*z* range of 150–1000, and the resolution was set to 140,000 mode. Flow injection analysis was done with a sample loop approach, injecting 80 μL and flowing at 20 μL min^–1^ with ultrapure 50:50 methanol/H_2_O, collecting data for 3.5 min (∼400 transients), similar to ‘direct infusion’ mode. Spectra were calibrated with common DOM ions as described previously,^6^ and formulas were assigned with an in-house MATLAB code (available in the Supporting Information), with elemental constraints as follows: C: 4–50, H: 4–100, O: 2–40, N: 0–2, and S: 0–1.

Samples were also analyzed on a 15T Bruker solariX XR Fourier transform ion cyclotron resonance mass spectrometer (FT-ICR-MS) equipped with an electrospray ionization (ESI) source (Apollo II), with analysis of the samples using an automated sample injector (CTC Pal3). Samples of deuterated internal standards were prepared in concentrations from 0.005 to 5 ppb either in ultrapure 50:50 methanol/H_2_O or in DOM samples with 5 mg L^–1^ DOM by mass in a 50:50 ultrapure methanol/H_2_O mixture. Thereafter, the mixtures were filtered through 0.2 μm PTFE filters and measured in broadband ESI negative ionization mode, with a flow rate of 6 μL min^–1^ and an ion accumulation time of 0.2 s. The capillary voltage was set to 4 kV. Two hundred scans were accumulated for each spectrum. To control the instrument drift, an in-house deep-sea reference material^30^ (NEqPIW, 2.5 ppm of DOC) was measured several times each day. Acquired mass spectra were calibrated internally against a NEqPIW-based or a contaminant-based mass list of confirmed molecular formulas with an error of <0.1 ppm. Afterward, the mass spectra were processed using the ICBM-OCEAN open access processing tool^31^ to remove noise and align samples along matching masses (tolerance 0.5 ppm). To further enhance mass precision, we used a recalibration function with elemental constraints as follows: C: 1–50, H: 2–80, O: 2–15, and N: 0–1. Molecular formulas were assigned after calibration using constraints as follows: C: 1–100, H: 2–200, O: 0–70, N: 0–4, S: 0–2, and P: 0–1 and also to the added synthetic products containing up to 5 deuterium atoms. The mass error, i.e., the difference between measured and known masses, was always <0.3 ppm. The method detection limit was set to 3.0 in ICBM-OCEAN.

### Response Factor Data for Isolated Acids

Spike compounds (Table 1) were prepared accurately to 1–1.3 mg mL^–1^ each and diluted together to a total mixture of exactly 1 mg L^–1^ of each compound. This was diluted 1:100 into the pure solvent (50:50 methanol/H_2_O) or 50 mg L^–1^ DOM sample matrix (river samples 1 and 2, coastal sample, or deep-sea sample in 50:50 methanol/H_2_O) to give a solution with 10 ppb of each compound. Each compound was also made to 10 ppb individually to check for adduct formation or in-source fragmentation and to test for ionization suppression when present in the mixture of compounds. The compound mixture was analyzed three times consecutively by the flow injection method described above for comparison with the unspiked pure solvent and the four DOM (which each had the same addition of 50:50 methanol/H_2_O added instead), and these unspiked matrices were also analyzed 3 times consecutively. The response factor of each acid was determined as the difference in the mean value of the deprotonated ion intensities per ppb with and without the spike in each solvent/matrix system.

**Table 1:**
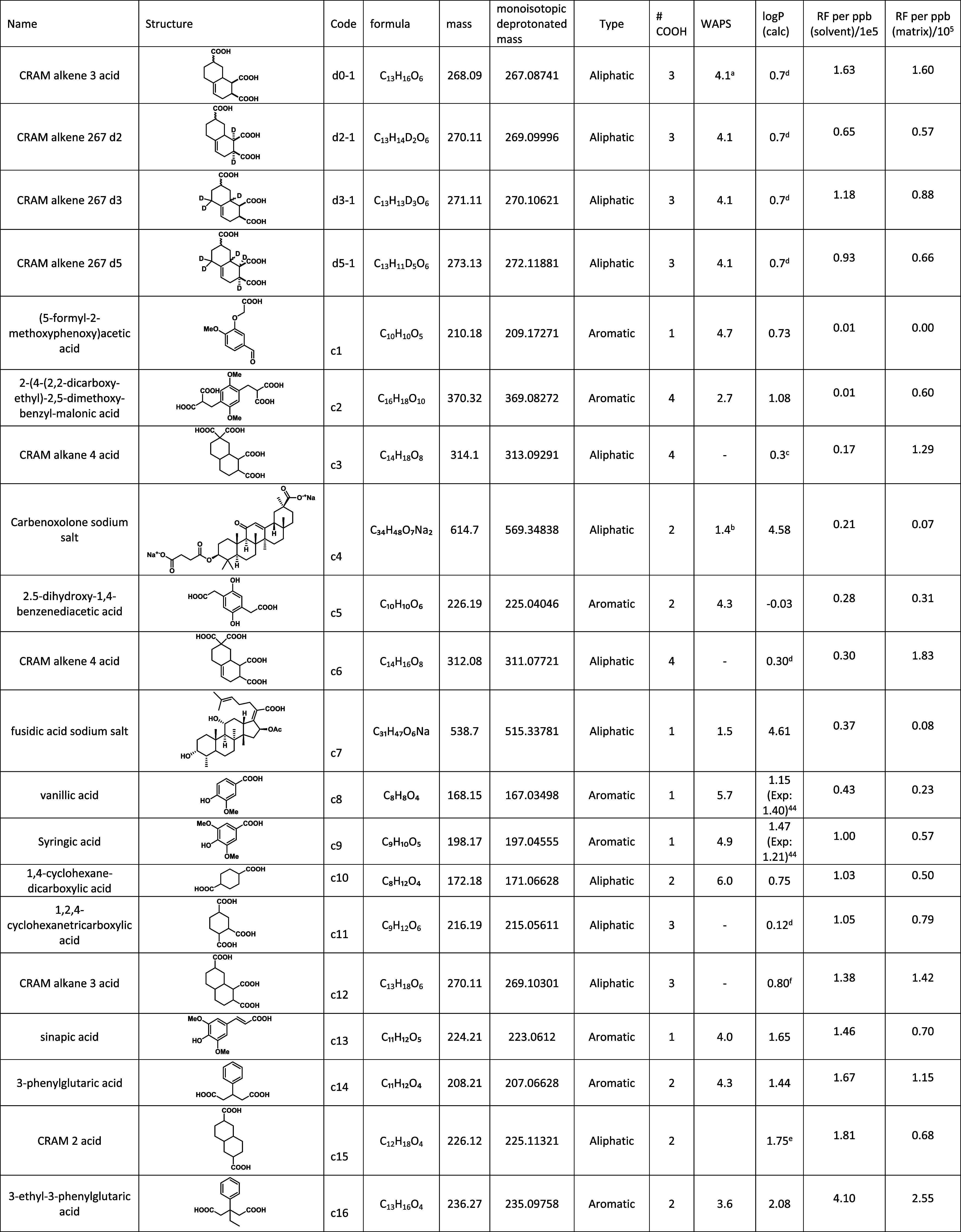
Compounds Purchased or Synthesized for Testing of Response Factors along with Calculated Physiochemical Data and Obtained Response Factors^a^

a Experimental RFs are calculated as the average of the response in the matrix averaged across TRM, SRFA, SRNOM, and NEqPIW.

b Charge localization (WAPS) calculated and averaged between two representative isomers.

c WAPS calculated for monoionic species.

d logP (calc) calculated using COSMO-RS and averaged between eight isomers.

e logP (calc) calculated using COSMO-RS and averaged between four isomers.

f logP (calc) calculated using COSMO-RS and averaged between six isomers.

g logP (calc) calculated using COSMO-RS and averaged between 16 isomers.

The response factor of the internal standard compound *d*_*5*_-**1** was used to pseudoquantify other peaks in the DOM samples analyzed. This was done by using the slope and intercept of the internal standard in the same mixture as a regression slope for each of the other assigned peaks, whose calculated quantities were then summed to estimate the total ionizable acid content of the sample.

### Liquid Chromatography Data

The 1000 mg L^–1^ stock of coastal DOM was diluted to 900 mg L^–1^ for LC-MS analysis, also with the addition of *d*_*5*_-**1mix** between 0.4 and 100 ppb. LC data was collected with the same instrumentation and settings as flow injection data, but by injecting 30 μL of the sample and running a 15 min reversed-phase gradient method (5–100% acetonitrile in water with 0.1% formic acid from 1 to 10 min) on a UPLC column (150 × 2.1 mm^2^, 2.6 μm C18, Kinetex, Phenomenex) set to 40 °C, with a flow rate of 500 μL min^–1^.

### Calculation of WAPS and logP Values for Carboxylic Acids

Conformer search for neutral and monoanionic forms of the compounds was carried out using COSMOconf software (version 24.0.1).^32^ In the case of polyacids, all protomers of monoanions were taken into account. In the case of compounds where the exact isomer was not known, several representative isomers were computed.

The molecular geometries were optimized at the DFT RI*-*BP86/def*-*TZVP level of theory with the COSMO model (*e* = ∞) using Turbomole V7.8 software.^33^ After that, single-point calculations were carried out at the RI*-*BP86/def2*-*TZVPD/COSMO level with the FINE cavity parameter. The results of these calculations—geometries, distributions of partial charges on molecular surfaces, and energies of the molecules in an ideal conductor—are the input for the following steps.

LogP values (logarithms of the partition coefficients of neutral forms of the compounds between mutually saturated *n-*octanol and water) were computed using the COSMO-RS^34–36^ method implemented in COSMOtherm software (release 2024, parametrization BP_TZVPD_FINE_24). All previously found conformers of neutral species were automatically taken into account by weighing according to their calculated relative energies in the liquids in question.

The WAPS (weighted average positive sigma)^37^ values for the monoanions were calculated from the partial charge distributions on molecular surfaces (eq 1)­

1 $$\mathrm{WAPS}=\frac{\sum_{\sigma >0}\sigma_{i}\cdot A_{i}}{\sum_{\sigma >0}A_{i}\cdot \sum_{i}A_{i}}\cdot 10^{5}$$

where *A*_i_ denotes the area of the surface segment i, and σ_i_ denotes the charge density (charge/area ratio) of the segment i. The WAPS values of individual conformers were weigthed according to their relative abundances in water, calculated by COSMOtherm.

## Results and Discussion

### Performance of *d_2_*-**1**, *d_3_*-**1**, and *d_5_*-**1mix** as Internal Standards

Three deuterated internal standards were evaluated for resolution from complex coastal DOM (TRM-0522 standard) using the LC-MS mode of analysis using QE Orbitrap MS (*d*_*2*_-**1**, *d*_*3*_-**1**, *d*_*5*_-**1mix**, Figure 1B) and with direct infusion using 15T FT-ICR-MS (Figure 1C, one example shown). Of the three, only *d*_*5*_-**1mix** was clearly separated from the existing ion peaks in coastal DOM using QE Orbitrap MS (nearest peak +3.28 mDa measured apex difference). *d*_*2*_-**1** was on the shoulder of a large peak (+2.98 mDa measured apex difference), and *d*_*3*_-**1** almost completely overlapped an existing ^13^C-containing isotopologue peak for the equivalent formula with one extra saturation (i.e., C_12_H_17_O_6_^13^C; + 0.18 mDa theoretical mass difference), with this split being *d*_3_ vs ^13^CH_2_ (Figure 1C). FT-ICR-MS was easily able to resolve *d*_*2*_-**1** and *d*_*5*_-**1mix**, and it partially resolved the 0.18 mDa split of the *d*_*3*_-**1** compound (Figure 1C).

The response slope of the two obtained isomers of *d*_*5*_ **-1** (*d*_*5*_ **-1a** and *d*_*5*_ **-1b**, and also *d*_*5*_ **-1mix**) at different concentrations in coastal DOM differed slightly, with *d*_5_-**1b** (the more hydrophobic isomer by LC-MS) having a lower response relative to a prominent DOM peak (*m*/*z* 367.1398; Figure SI1). This result was quite unexpected, but the mechanisms governing the relative ESI response factor are still not fully understood, despite several studies attempting to build multivariate models for this purpose,^15,38,39^ with investigations or explanations of stereochemical effects currently underdeveloped.^39^

The internal standard *d*_*5*_-**1a** was added at 10 ppb to different concentrations of coastal DOM between 5 and 75 ppm of the total material and measured by QE Orbitrap MS. Because the *d*_*5*_-**1a** mass spectral peak had no interference in the complex mixture (Figure 1B), it was possible to use its response as an internal standard at differing concentrations of coastal DOM, reporting all other intensities as a ratio of this peak (i.e., dividing the intensity by the intensity of the internal standard) (Figure 2B). Applying this normalization dramatically improved the linearity of response of all major peaks in the complex mixture, apparently countering nonlinearity effects previously observed for single compounds.^40^ For intense peaks such as *m*/*z* 367.1398, the normalization lead to an an *r* ^2^ value of 0.997 (Figure 2B), alleviating the effects of both ionization suppression (loss of linearity) and replicate differences (imprecision on the *y*-axis). Assessment across nearly 1500 peaks in coastal DOM shows that the *r* ^2^ value was close to 1 for most highly responding ions. Specifically, 93.8% of peaks >1% of the maximum height had an *r* ^2^ value >0.95 after normalization (Figure 2C). These results indicate that an internal standard such as *d*_*5*_-**1** can greatly assist in pseudoquantification efforts in DOM studies,^41^ because it provides a drastic improvement in linearity in almost all of the DOM peaks without interfering with the mass spectrum and can be used at very low spike amounts (1:5000 in the case of the 10 ppb spike in 50 ppm of DOM), therefore providing very little ionization suppression itself.

**Figure 2:**
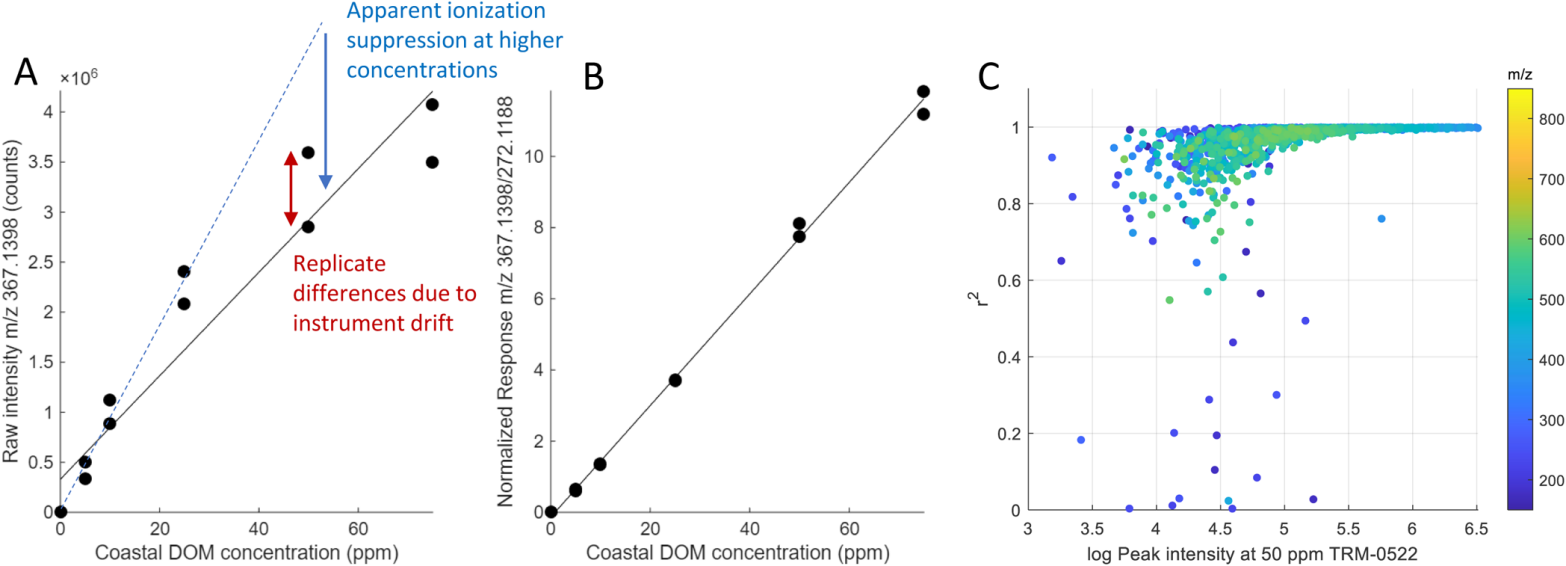
Calibration series and linear regression of the highest-responding ion in coastal DOM as the coastal DOM concentration increases, measured by QE Orbitrap MS. (A) Raw data (*r* ^2^ = 0.909), with a blue dotted line showing linearity from the first three concentrations and (B) normalized to a 10 ppb addition of *d_5_*-**1a** (*r* ^2^ ^2^ = 0.997). (C) Linearity (*r* ^2^) of the response normalized to *d_5_*-**1a** of all formulas that were found in coastal DOM at all concentrations (5–75 ppm) (a total of 1459 formulas); note the log scale of the *x*-axis.

### Evaluation of DOM Sample Ionization Suppression Using the *d_5_*-**1a** Internal Standard and Comparability between Instruments

Calibration slopes for *d*_*5*_-**1a** were prepared by serial dilution in the pure solvent and three DOM reference materials at a 50 ppm concentration. For Orbitrap data, the response was linear in all cases (*r* ^2^ > 0.998), and the response factor (regression slope) was much lower in the DOM samples (40–50%) compared to the pure solvent (Figure 3A). This decrease in the response factor was presumably due to ionization suppression, which does typically decrease the intensity of response of various metabolites to about 50% in negative mode for DOM samples.^42^

**Figure 3:**
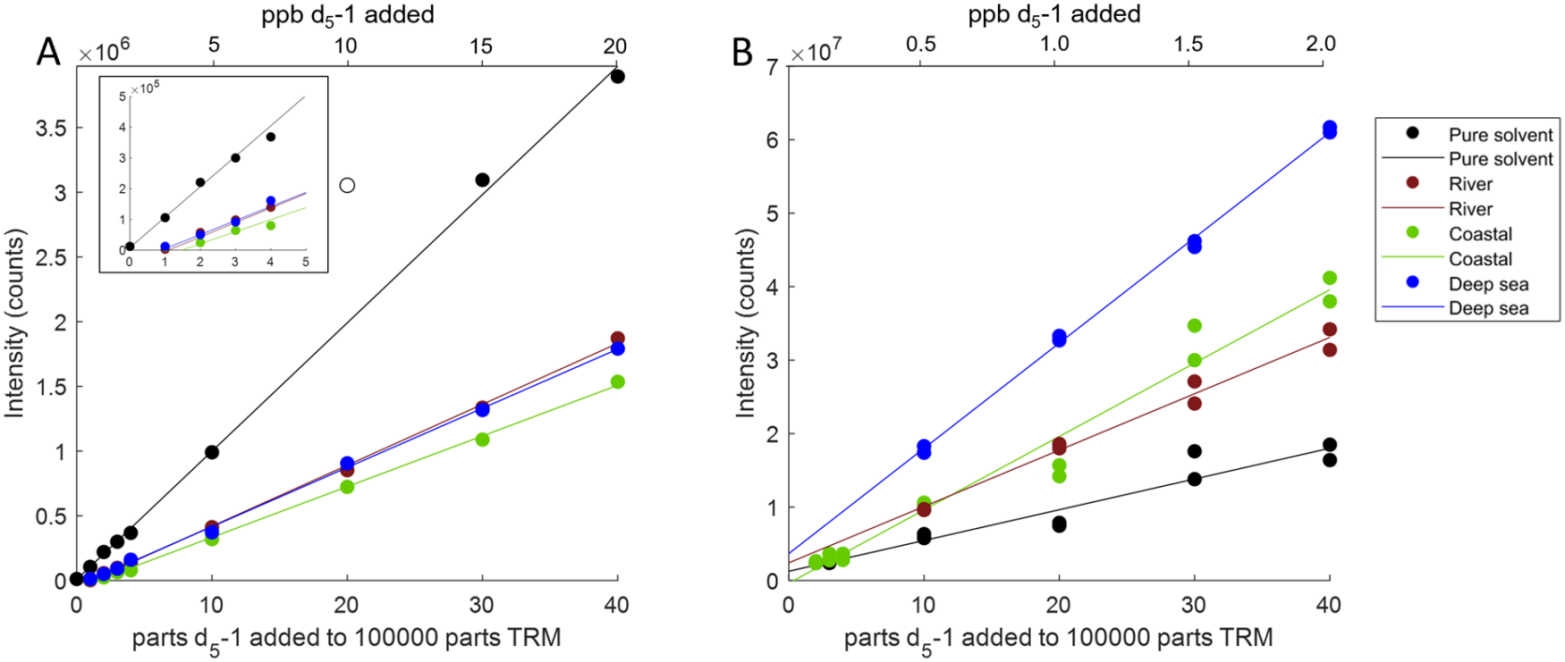
Response of *d_5_*-**1a** in four different sample matrices in flow injection mode (A) for QE Orbitrap MS and (B) for 15T FT-ICR-MS. The inset in panel (A) shows an expanded view of the area close to the origin. Note one outlying point for the pure solvent matrix at 20 parts/1,00,000 that was excluded from the regression, shown as an unfilled symbol in panel (A). The concentrations (both for the internal standard and the matrix) were 10× lower for FT-ICR-MS due to the higher sensitivity of the instrument (see the upper *x*-axis). In FT-ICR-MS software processing, transients are added, whereas they are averaged in Orbitrap, which partly explains the magnitude difference in signals in the *y*-axis.

The calibration graphs did not include the data collected at the zero addition of *d*_*5*_-**1a**, which would have forced the lines closer to the origin. Instead, the position that the graphs pass through the *x*-axis is meaningful because it indicates that the detection limit in concentration in the DOM sample matrix is about 0.5 ppb on Orbitrap (Figure 3A, inset), which is much higher than in the pure solvent. The reason for the nonzero cutoff in the *x*-axis for the complex mixtures is due to the trap capacity and post-Fourier transform zero-filling algorithms (‘reduced-profile mode’) for Orbitrap.^43^ This decreases the dynamic range of the instrument, cutting off true signals that are close to noise because they do not benefit from the multiplexing effect of coadding fully noised spectra.

Using 15T FT-ICR-MS, we were surprised to find that *d*_*5*_-**1a** responded better in the DOM matrix compared to the pure solvent. Moreover, the signal response was the highest in the deep-sea DOM sample compared with the coastal and river matrices, likely due to the standard tuning procedure of 15T FT-ICR-MS in question using the deep-sea DOM sample (NEqPIW) for signal optimization.^44^ Using FT-ICR-MS, we analyzed *d*_*5*_-**1a** at a 10× lower concentration in a 10× more dilute matrix due to the higher sensitivity of the instrument and obtained data with slightly less linearity and precision compared with QE Orbitrap MS. The lower linearity and precision may be due to the setting of the trapping time at 0.2 s for FT-ICR-MS, while Orbitrap uses automatic gain control, allowing an automated higher trapping time for more dilute samples. Overall, FT-ICR-MS appeared to have slightly lower precision and more sensitivity to the matrix type, suggesting that FT-ICR-MS would benefit even more from the inclusion of an internal standard than Orbitrap. Note that neither instrument was specifically optimized or tuned for sensitivity at the mass in question (272 Da), nor was the detection limit formally investigated. Both are likely to be improved for the detection of a specific compound using selected ion monitoring prior to data acquisition, but the purpose of the internal standard is rather to be used during full mass range transients for normalization of other peaks, such as that shown in Figure 2.

### Evaluation of the Response Factor for Multiacids in Comparison to *d_5_* **-1**

Several acids were purchased or synthesized to compare their relative response factors (i.e., measured ion intensity per concentration) by flow injection ESI-Orbitrap analysis (Table 1). The purpose of this experiment was to determine whether their response factors could be modeled based on chemical properties^15,38^ and to evaluate an average response factor for carboxylic acids for comparison with *d_5_*-**1** and for use in the modeling concentration of compounds contributing to DOM mass spectra.

The 20 compounds tested exhibited varying response factors with an interquartile range of 0.3–1.4 × 10^5^ counts/ppb. This range is considerably smaller than in some studies,^15,39^ which span orders of magnitude due to a much greater structural diversity. In contrast, the compounds selected here were chosen to closely represent the structural features proposed in DOM and have a limited range of physicochemical properties, such as logP (Figure SI2 and Table 1). Easily the highest-responding acid was 3-ethyl-3-phenylglutaric acid, with a value of 4.1 × 10^5^ counts/ppb. The mean response factor was 0.98 × 10^5^ counts/ppb (a median of 0.96 × 10^5^ counts/ppb). In the matrix (averaged across the four matrices), the counts/ppb were always lower, with a mean and a median of 0.82 × 10^5^ counts/ppb and 0.67 × 10^5^ counts/ppb, respectively (Figure 4).

**Figure 4:**
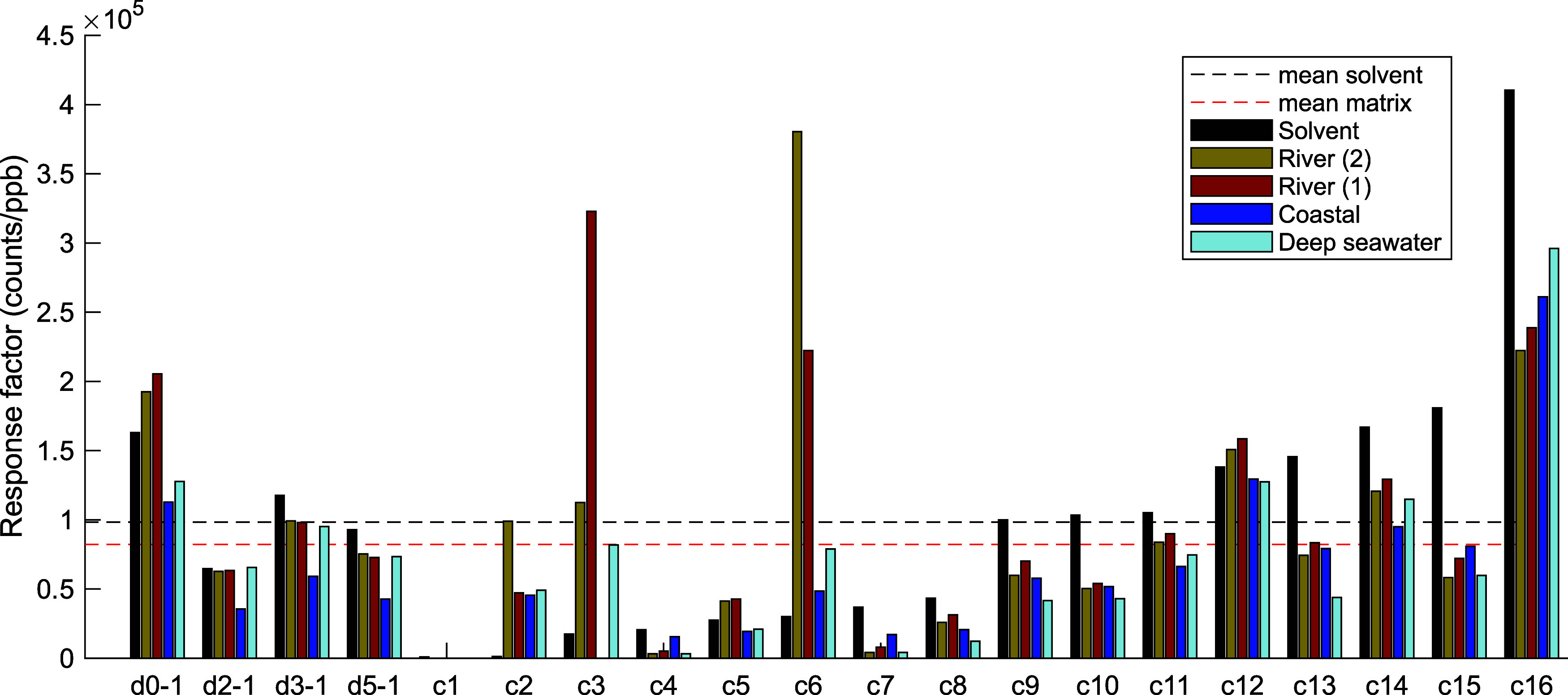
Response change for 20 different acids per part per billion addition in pure 50% methanol or in 50 ppm of the matrix (see the legend) as measured by QE Orbitrap MS. The mean of the 20 acids is shown as a dashed line for pure 50% methanol (black) or as the average of the four matrices (red).

The 20 standards can be categorized into low-responding (**c1**, **c4**, **c5**, **c7**, **c8**), medium-responding (*d_0_*-**1**, *d_2_*-**1**, *d_3_*-**1**, *d_5_*-**1**, **c9**–**c15**), high-responding (**c16**), and erratic (**c2**, **c3**, **c6**) types as measured by QE Orbitrap MS. The low-responding compounds typically had features that would not be considered normal for marine DOM; for example, **c1** has a phenolic ether linkage to the acid group, which may be labile to fragmentation under ESI spray; vanillic acid (**c8**) has a very low mass (*m*/*z* 167); and **c4** and **c7** are very hydrophobic compared to DOM (Table 1). The high-responding compound, 3-ethyl-3-phenylglutaric acid (**c16**), is slightly unusual in that it has a central quaternary carbon with two hydrophobic and two hydrophilic groups, which may give an overall polarity that is especially well suited to ESI. The behavior of the ‘erratic’ group is difficult to explain and includes cases where the response was very low in the pure solvent but very high with the DOM matrix. These compounds all contain four carboxylic acid groups and include two synthesized ‘CRAM-like’ compounds with a 1,1-diacid functionality and a purchased compound with 1,1-diacid functionalities (**c2**). Notably, we have found compounds **c3** and **c6** to be labile to in-source fragmentation (single neutral loss of CO_2_) under ESI during their initial isolation, which may contribute to their erratic behavior in different matrices. The medium-responding group, accounting for 11 of the 20 compounds, included all of the examples of alicyclic carboxylate-rich molecules except for the 1,1-diacids, which would suggest that DOM responds similarly to these medium-responding compounds, under the assumption that DOM is constituted by such aliphatic multiacids.^45^

It is not a surprising result that different acids have different response factors or that it is challenging to model the reasons why certain acids respond better than others to ESI.^15^ In complex mixture analysis, when the analyte identity is unknown and each ion peak observed is a mixture of isomers, the data obtained can be still used to estimate concentrations due to the central limit theorem,^46^ stating that any mixture of isomers with differing response factors will yield a result close to the abundance-weighted average value of the mixture. This means that if we assume that the selected acids (particularly, the medium-responding ones) are representative of DOM acids, then the average response factor of the analyzed compounds can be used to model the response of the unknown DOM compound mixtures.^14^ This assumption may or may not be completely valid and certainly has a key unprovable assumption that the acids selected are representative in terms of the mean, variance, and skewness of structures really present in DOM, which is rather unlikely. However, any extreme values in the DOM mixtures will quickly be averaged away by opposing values, unless the mixture is very homogeneous and high- or low-responding, which we consider unlikely. In other words, because each of the observed peaks in DOM is a mixture of isomers, the signal response per ppb will tend toward a uniform value across the whole mass spectrum, and each peak can be pseudoquantified assuming the same response factor throughout.

A key question arises with regard to which value should be used for such an assumed average response factor going forward. Ideally, a much larger and provably representative test set of carboxylic acids could be used in all cases, but this is practically very challenging to achieve consistently, both within studies and among research groups. Instead, we propose that the *d*_*5*_ **-1mix** standard, which has a reasonably average response (see Figure 4) and can be separated from adjacent peaks in MS analysis, even at the modestly high resolving power obtained by Orbitrap (see Figure 1B), can be used for pseudoquantification. Alternative compounds would be **c9**, **c11**, **c13, c14**, or **c15**, which have responses close to the average and are reasonably consistent between different matrices. However, all of these compounds have isobaric or isomeric interferences in DOM, making them less attractive internal standards than *d*_*5*_ **-1**.

In this case, we used the response of *d*_*5*_-**1a** for the pseudoquantification of other peaks in the four DOM samples measured using QE Orbitrap MS and 15T FT-ICR-MS using the calibration slopes shown in Figure 3. In samples (from Figure 3) which had 10 ppb of *d*_*5*_-**1a** and 50 ppm of DOM for Orbitrap and 1 ppb of *d*_*5*_-**1a** and 5 ppm of DOM for FT-ICR-MS, all of the other peaks that had formulas assigned were pseudoquantified to ppb values with the *d*_*5*_-**1a** calibration slope. These ranged between 0.75 and 320 ppb for the coastal reference sample TRM on Orbitrap, with the 320 ppb peak being an outlier more than twice as intense as the next most intense peak. As listed in Table 2, the 10th and 90th percentile peak quantities are shown (in ppb), along with the sum of all peaks (in ppm), which are compared with the actual ppm concentration of the mixture analyzed as a metric ‘% acids’.

**Table 2:** Pseudoquantification (on a Mass Basis) of Peaks in Four DOM Samples Using the Compound *d_5_*-**1a** as an Internal Standard

	QE orbitrap MS (solution conc. 50 ppm)				15T FT-ICR-MS (solution conc. 5 ppm)			
DOM sample	10th percentile (ppb)	90th percentile (ppb)	sum (ppm)	% ionizable acids	10th percentile (ppb)	90th percentile (ppb)	sum (ppm)	% ionizable acids
TRM	0.76	7.12	15.1	30.2	0.02	1.52	0.92	18.5
SRFA	0.57	8.06	10.7	21.3	0.09	1.26	0.56	11.1
NEqPIW	0.46	4.59	10.4	20.7	0.10	3.04	1.19	23.8

The estimated concentration values of 4233 peaks assigned to formulas in coastal DOM at percentiles 10, 50, 90, and 99 were 0.77, 1.35, 7.12, and 40.3 ppb (i.e., μg/L on a mass basis), respectively, showing that the abundances are heavily skewed toward lower values. The sum of all calculated ppb values of the peaks in the data set equaled 15.1 ppm, which is reasonably close to the prepared 50 ppm value, and taken at face value, suggests that around 30% of this sample is composed of acids that ionize like the compound *d*_*5*_-**1a**. The lower than 100% ‘recovery’ can easily be accounted for by the presence of nonacid materials in the sample that gives a lower response in negative-mode ESI,^14^ unassigned formulas, and the bias of the ESI-MS response toward a certain mass window. Unassigned peaks accounted for ∼5, 4, and 17% of the intensity for coastal, river, and deep-sea DOM, respectively, for Orbitrap, suggesting that little ionizable material was left unaccounted for. However, the mass window that the instrument is sensitive to and accurate over never completely captures the whole DOM sample and can vary significantly between different instruments according to how they are tuned.^6^

In the other two DOM samples pseudoquantified the same way, summed peak intensities were slightly lower at approximately 10 ppm. Using FT-ICR-MS, similar values were obtained for ‘% acids’ for the deep-sea reference sample from NEqPIW, but the river reference sample SRFA and coastal reference sample (TRM) gave somewhat lower values. This may be due to the combination of the differing responses of *d*_*5*_-**1a** in this instrument for the different DOM matrices (Figure 3B) and the fact that this particular instrument has generally been carefully tuned for the best response in marine samples, while Orbitrap software and instrumentation allow a less extent of custom tuning. Unassigned peaks accounted for <3% of the intensity for FT-ICR-MS. Overall, these results reiterate the fact that different high-resolution ESI-MS instruments can give somewhat different and only semiquantitative results^6^ and that using more internal standards to represent different types of compounds is likely to be preferable for quantification.^47^ Either way, having at least one internal standard seems to be critical if any type of intersample peak response comparison is to be attempted.

## Compound Detection Limit in LC-MS Mode

The diastereomeric mixture *d*_*5*_-**1mix** was spiked into 900 ppm of coastal DOM at concentrations ranging between 0.4 and 100 ppb, and the resulting mixtures were analyzed on a gradient LC-MS method using C18 as the stationary phase. The calibration was extremely linear, with a slope and an *r* ^2^ value of 1.40 × 10^5^ and 0.998, respectively, from 0 to 20 ppb and 1.46 × 10^5^ and 1.000 from 0 to 100 ppb, respectively. The formal detection limit based on the calibration of *d*_*5*_-**1mix** over 0–20 ppb (using LOD = 3.3 × standard error in intensity/slope + intercept in concentration) was 1.57 ppb, equivalent to 1.74 ppm of the injected DOM mixture (which was 900 mg L^–1^). SPE-DOM has a concentration in the deep ocean of around 27 μM (or 0.3 mg L^–1^) C, and marine DOM is approximately 50% carbon,^30^ so deep-sea SPE-DOM concentrations are about 0.6 mg L^–1^. This, combined with the limit of detection obtained, suggests that any individual acid compounds could be detected as a chromatographic peak as low as 1.04 ng L^–1^ in solid-phase-extracted seawater. Considering that the detected signals are still unresolved in a chromatographic sense, the true compound concentrations must still be lower than this.

The detection limit was approximately 20× higher in flow injection (direct infusion) mode because the analytical detection limit was similar (about 1.5 ppb), but the concentration of bulk DOM was 18× lower (50 vs 900 ppm). This means that samples could be concentrated approximately 20× less in the flow injection mode, and this demonstrates the value of LC-MS mode in trace compound discovery along with the improvements it gives in the investigation into DOM isomerism.^17,41,48,49^ Detection limits could be lowered further with prior fractionation of SPE-DOM samples,^25,50–52^ with selected ion monitoring, with derivatization, or through improved chromatographic resolution. However, current Orbitrap technology and the limits imposed by complex mixture analysis and limited ion trap capacity mean that approximately 1 ng L^–1^ is a reasonable rough approximation for the detection limit of carboxylic acids in a nontargeted LC-MS approach from a seawater extract. Extracting higher volumes of water cannot improve this detection limit greatly unless the sample is fractionated, due to the limits imposed by ionization suppression and trap capacity.

Figure 5 shows the robust retention time and peak shape determined for the major isomer of *d*_*5*_-**1** when added to a concentrated TRM solution, while other peaks in the mixture (which elute very broadly, as is well known for DOM) are unaffected by the addition. The analysis conditions were identical to those of the direct infusion source, but the flow rate was much higher and the overall coastal DOM concentration was 18 times higher, despite the same calibration range for the compound. Due to the high efficiency separation using UPLC elution, the peak width at half height was only about 1.8 s, only allowing about 7 transients over the whole peak, showing that the transient time required for the resolution of *d*_*5*_ **-1** is not fast enough for ideal chromatographic peak characterization at the best performing chromatographic conditions. Slower separations may help here, but at the cost of chromatographic resolution, which is a key objective in ongoing DOM structural characterization research.

**Figure 5:**
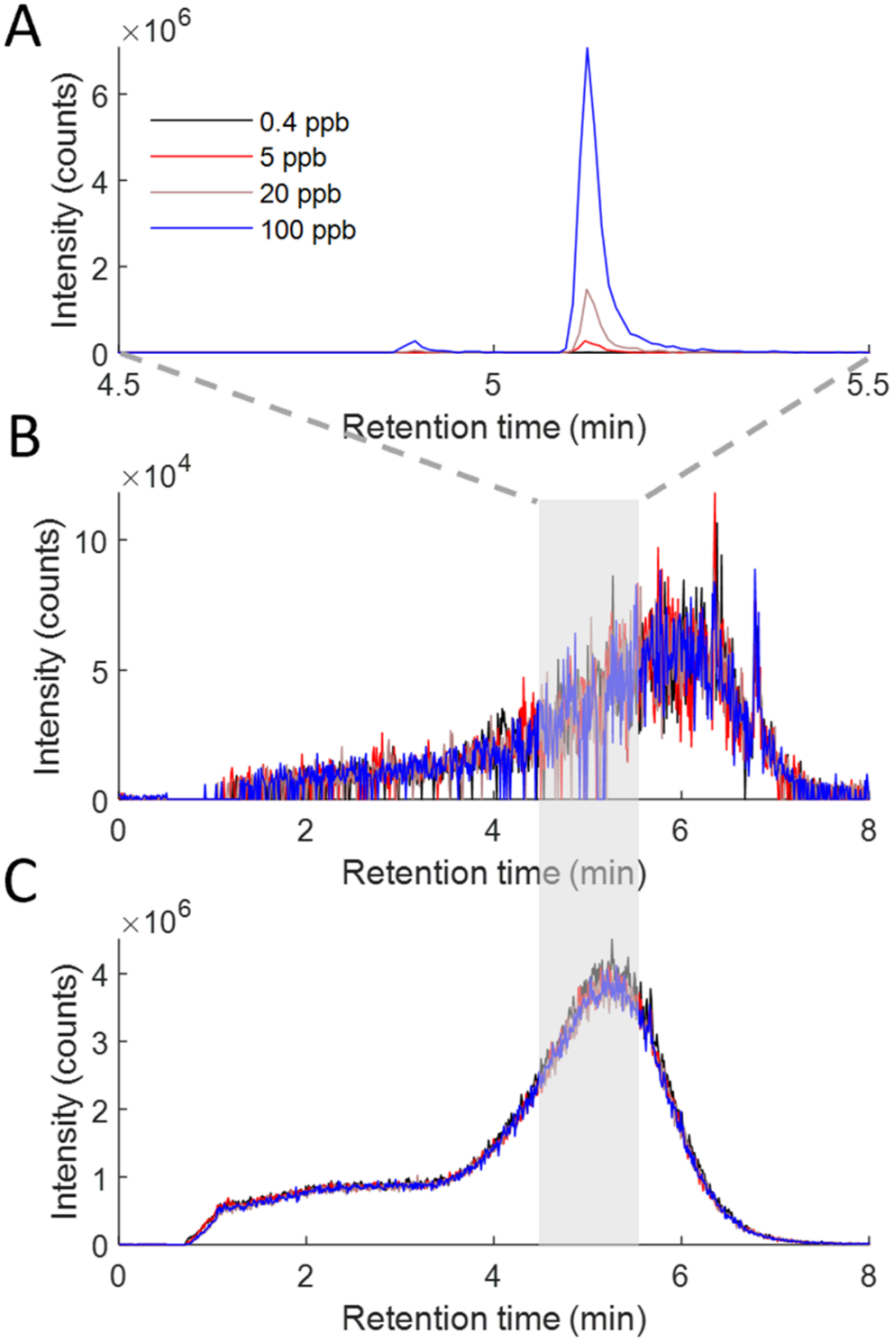
LC-MS mode data: (A) *d_5_*-**1mix**, (B) the adjacent peak in TRM at *m*/*z* 272.12155 (Figure 1), and (C) the highest peak in TRM (397.118), all shown at four spike concentrations of *d_5_*-**1mix** (0.4, 5, 20, and 100 ppb). The top panel shows that the retention time and peak shape do not vary with the increasing concentration, while the bottom two panels show that an adjacent peak and the highest peak are unaffected by the changing concentration of the spike. Note the different *x*- and *y*-axis scales.

## Conclusions

We have shown that deuterated CRAM-like compound standards can serve as useful internal standards for DOM research. The *d_2_* and *d_3_* isotopologues (*m*/*z* 269 and 270) are very challenging to resolve from isobaric interferences in DOM using Orbitrap, but resolution is achievable with 15T FT-ICR-MS, particularly for the *d_2_* example. The *d_5_* compounds can be resolved with both methods (at *m*/*z* 272) and are therefore more universally applicable across platforms. The use of a small spike of a deuterated internal standard (e.g., 10 ppb in 50 ppm of DOM) allows factors like ionization suppression and instrument drift to be accounted for, vastly improving the linearity of other peaks with the increasing concentration. In principle, the use of an internal standard also allows “pseudoquantification”, under the assumption that the peak of interest is composed of compounds that have a similar response factor to the internal standard. We found that *d*_*5*_-**1mix** had a response factor quite close to the average of 20 acids with DOM-like features and so propose that it is a suitable internal standard for such purposes.

Using this pseudoquantification method, we determined that TRM coastal marine reference DOM contains approximately 30% acids that ionize like *d*_*5*_-**1**, while SRFA has around 21%, as determined by QE Orbitrap MS. However, results were different using 15T FT-ICR-MS, presumably due to different ESI and instrument optic tuning, leading to estimates of 19 and 11% acids, respectively.

We also determined that our standard LC-MS method would have a feature detection limit of about 1 ng L^–1^ DOM compound in seawater, again assuming a similar response factor to the *d*_*5*_-**1mix**, and we recommend the use of a *d*_5_-labeled standard (for example, infused post-column to correct for solvent gradient effects)^41^ for any such quantification of features using LC-MS analysis in future work.

## Supplementary Material





## Notes

Additional figures; extended description of synthetic methods; and general information about chemical synthesis including reaction schemes and all ^1^H NMR spectra of synthesized compounds and intermediates (PDF)
Raw data files from LC-HRMS and MATLAB script with all data processing steps (ZIP)
