# Supplementary material for: Improvement of Electrospray Ionization Response Linearity and Quantification in Dissolved Organic Matter Using Synthetic Deuterated Internal Standards
Source: Anal Chem. 2025 Aug 22;97(34):18562–72. doi: 10.1021/acs.analchem.5c02463 (PMC12409698; doi:10.1021/acs.analchem.5c02463)
Supplement: Supplementary file 1 [file ac5c02463_si_001.pdf]

Supporting Information

Improvement of Electrospray Ionization Response Linearity and Quantification in Dissolved Organic Matter using Synthetic Deuterated Internal Standards

Alexander J. Craig[1,2], Mustapha A. Ganiyu[1,3], Lindon W.K. Moodie[2], Sofja Tshepelevitsh[3], Koit Herodes[3], Heike Simon[4], Thorsten Dittmar[4,5], and Jeffrey A. Hawkes[1]

1. Analytical Chemistry, Department of Chemistry BMC, Uppsala University, Uppsala, 75237, Sweden

2. Drug Design and Discovery, Department of Medicinal Chemistry, Uppsala University, Uppsala 75237, Sweden

3. Institute of Chemistry, University of Tartu, Ravila 14a, 50411, Tartu, Estonia

4. Marine Geochemistry, Institute for Chemistry and Biology of the Marine Environment (ICBM), Carl von Ossietzky Universität Oldenburg, Oldenburg, Germany

5. Helmholtz Institute for Functional Marine Biodiversity (HIFMB) at the Carl von Ossietzky Universität, Oldenburg, Oldenburg, Germany

*Corresponding author: jeffrey.hawkes@kemi.uu.se

Contents:

S2. Figure SI1: *Response of d_5-**1a** compared with d_5-**1b** and d_5-**1mix**. All three measured and plotted in duplicate as a relative response to the DOM peak 367.1398.*

S2. Figure SI2: *Response factor in pure solvent (left) and in matrix (averaged between 4; middle) for the 20 compounds, plotted vs calculated logP, followed by a comparison of the two, with a 1:1 line drawn. Synthesized 'CRAM-like' compounds are shown in red, and purchased compounds shown in black. The average response is lower in matrix, due to ionization suppression.*

S3. General Methods

S3. NMR Analysis Information

S4. General Synthetic Information

Supplementary figures

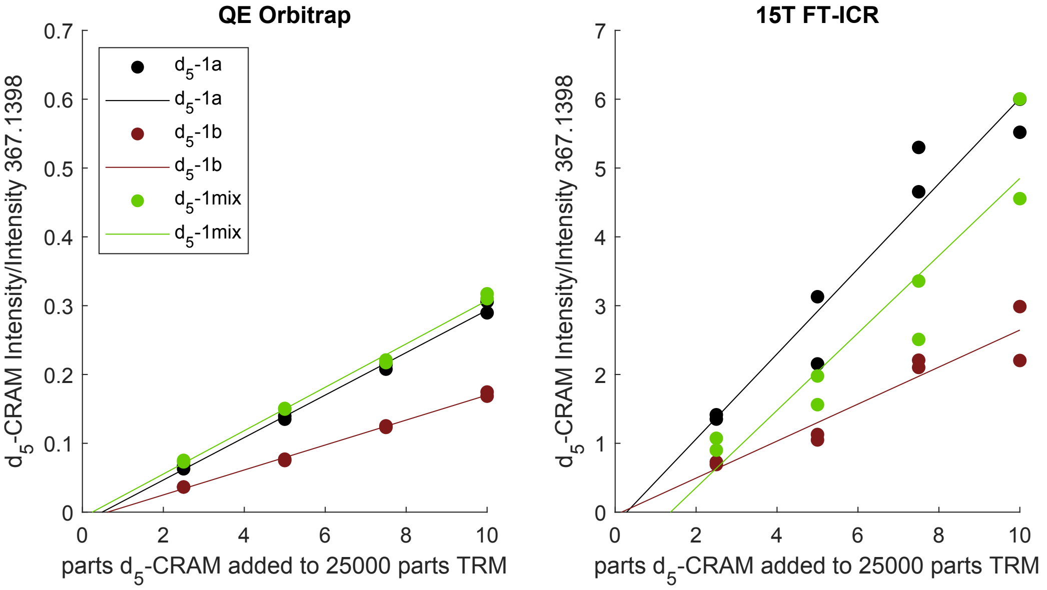

Figure SI1: *Response of d_5-**1a** compared with d_5-**1b** and d_5-**1mix**. All three measured and plotted in duplicate as a relative response to the DOM peak 367.1398.*

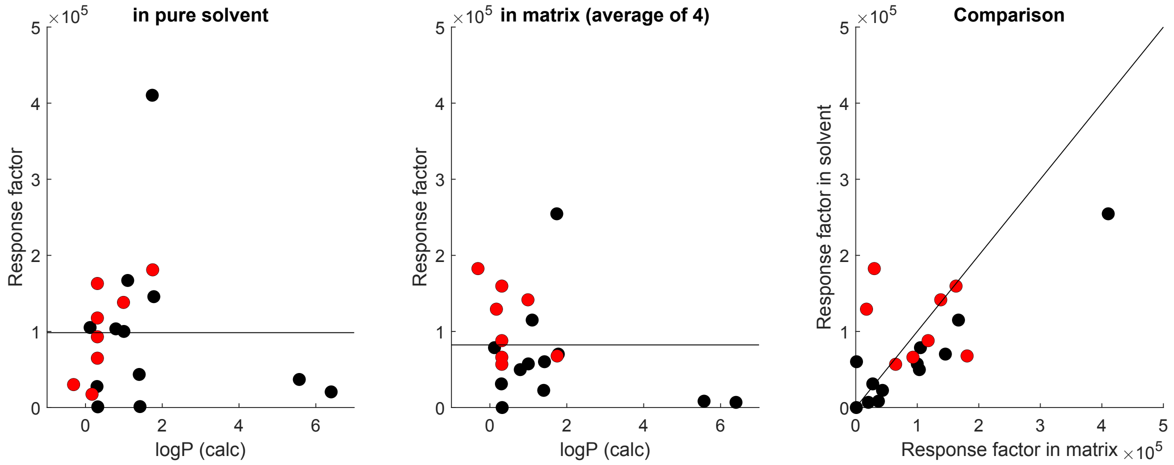

Figure SI2*: Response factor in pure solvent (left) and in matrix (averaged between 4; middle) for the 20 compounds, plotted vs calculated logP, followed by a comparison of the two, with a 1:1 line drawn. Synthesized 'CRAM-like' compounds are shown in red, and purchased compounds shown in black. The average response is lower in matrix, due to ionization suppression.*

General Methods

Reactions were monitored either by thin-layer chromatography (TLC), or with liquid chromatography mass spectrometry (LCMS). TLC was performed on 0.2 mm aluminium plates precoated with silica gel 60 F_{254} (Merck). Compounds were visualized with an ultraviolet (UV)-light, and stained with potassium permanganate. Column chromatography was performed with silica gel (40 – 63 μM). ^{1}H NMR spectra were recorded at 400 MHz on a Varian Mercury Plus spectrometer or at 500 MHz on a Bruker Avance Neo spectrometer with a TXO (CRPHe TR- 13 C/ 15 N/ 1 H 5mm-Z) probe at room temperature. All spectra were recorded from samples in either $CDCl_3$ or MeOD, at room temperature in 5 mm nuclear magnetic resonance (NMR) tubes. Chemical shifts are reported relative to the residual solvent peak at δ 7.26 for $CDCl_3$ or δ 3.31 for MeOD. Resonances were assigned as follows: chemical shift (multiplicity, number of protons, coupling constant(s)). Resonances were assigned as follows: chemical shift (multiplicity, number of protons, coupling constant(s)). Multiplicity abbreviations are reported by the conventions: s (singlet), dd (doublet of doublets), ddd (doublet of doublet of doublets), dddd (doublet of doublet of doublet of doublets), t (triplet), tt (triplet of triplets), m (multiplet). Proton decoupled ^{13}C NMR spectra were recorded at 101 MHz on a Varian Mercury Plus spectrometer under the same conditions as for the ^{1}H NMR spectra, or at 126 MHz on a Bruker Avance Neo spectrometer with a TXO (CRPHe TR- 13 C/ 15 N/ 1 H 5mm-Z) under the same conditions as for the ^{1}H NMR spectra. Chemical shifts are reported relative to the residual solvent peak at δ 77.16 for $CDCl_3$ or δ 49.90 for MeOD. Preparative high-pressure liquid chromatography (HPLC) was performed using a VP 125/21 NUCLEODUR C18 HTec column (21 × 125 mm, 5 µm particle size, Machery-Nagel) with a flow rate of 25 mL/min. All solvents and reagents were used as received.

NMR Analysis Information

^{13}C NMR peaks for the deuterated acids are listed first for their major isomer, and second for all peaks above an arbitrary threshold. This is due to the CD_x peaks in the minor isomers being too low in intensity to reliably observe.

General Synthetic Information

Preparation of the three deuterated analogues began with preparation of d_4-ketone **S2** from ketone **S1**, through base mediated deuterium exchange in D_2O. Following this, synthesis followed our previously disclosed route,[1] with Diels-Alder steps using either d_2-maleic anhydride **S8**, or maleic anhydride **S9**, depending on the target product. Initially, we did not perform purification after triflate formation, instead only purifying once Stille coupling had provided diene **S6**. However, yields were very low over the two steps (below 5%), and purification of this diene was difficult and led to very high amounts of $SnBu_3OH$ contamination (several equivalents compared to the diene). By purifying after the triflate formation, yields were slightly increased to 10% over the two steps, and the amount of vinyl tributyl tin significantly reduced, (approximately 10% $SnBu_3OH$ contamination).

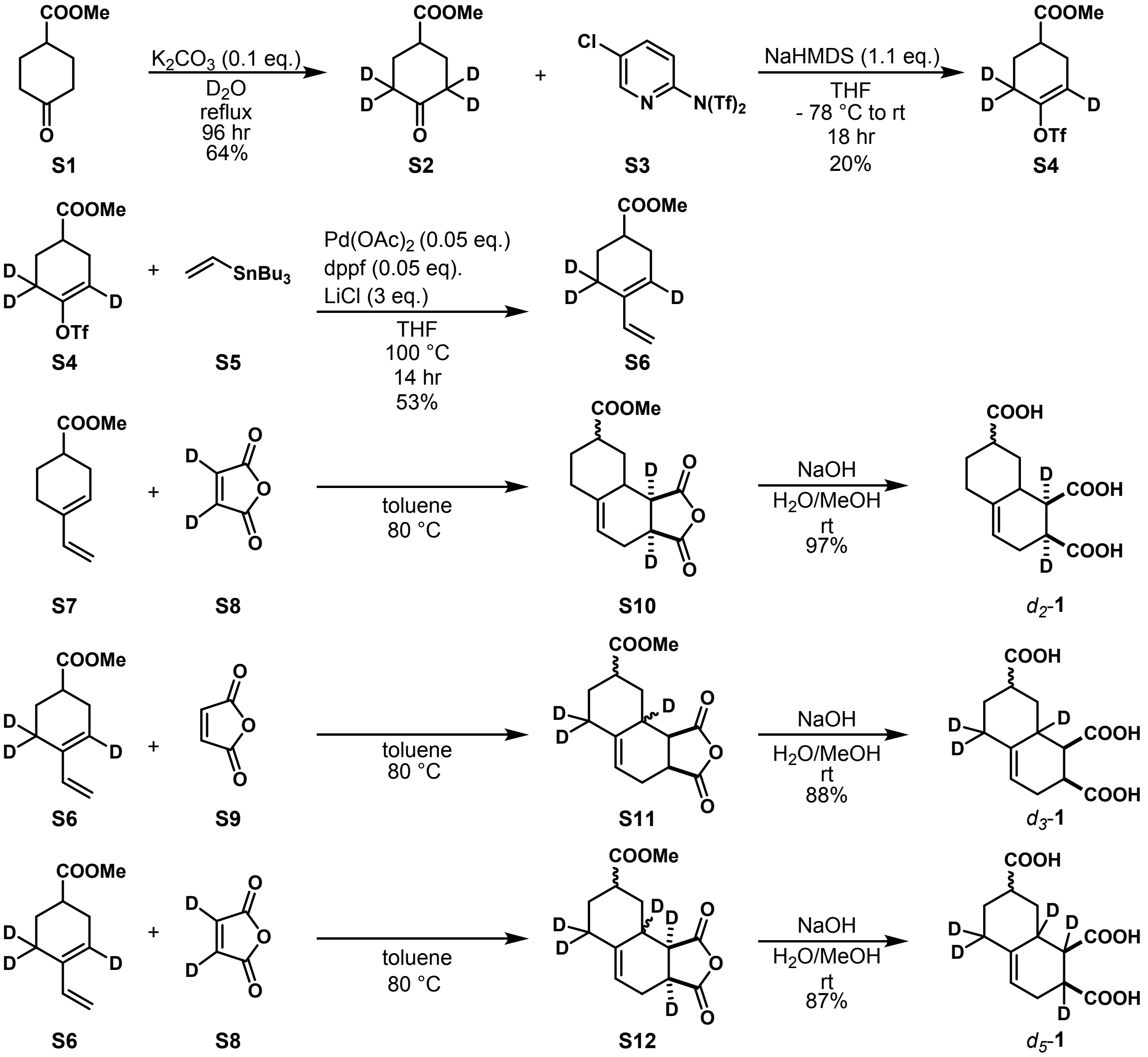

Scheme S1: *Synthetic route to prepare compounds d_2-**1**, d_3-**1**, and d_5-**1**.*

d_4-Ketone **S2**

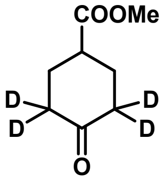

To methyl 4-oxocyclohexane-1-carboxylate (0.495 g, 0.45 mL, 3.17 mmol) was added D_2O (10 mL), before the mixture was heated to reflux and stirred for 4 days. At completion, the mixture was cooled, and extracted with Et_2O (x3). The organic portions were combined, and washed with distilled water (x2), and brine (x1), before being dried over Na_2SO_4, filtered, and dried in vacuo to afford the title compound as a pale-yellow oil (0.325 g, 64%).

^{1}H NMR (400 MHz, 298K, $CDCl_3$) δ 3.72 (s, 3H), 2.76 (tt, 1H, *J* = 9.8, 4.0 Hz), 2.19 (dd, 1H, *J* = 13.5, 4.0 Hz), 2.01 (dd, 1H, *J* = 13.4, 10.0 Hz).

^{13}C NMR (101 MHz, 298K, $CDCl_3$) δ 210.5, 174.8, 52.1, 40.7, 39.2 (t, *J* = 20.2 Hz), 28.6.

HRMS (ESI-MS) calculated for **S2** $C_8H_9D_4O_3^+$ $[M + H]^+$: 161.1110; not found.

^{1}H NMR spectra of compound **S2** (400 MHz, 298K, $CDCl_3$)

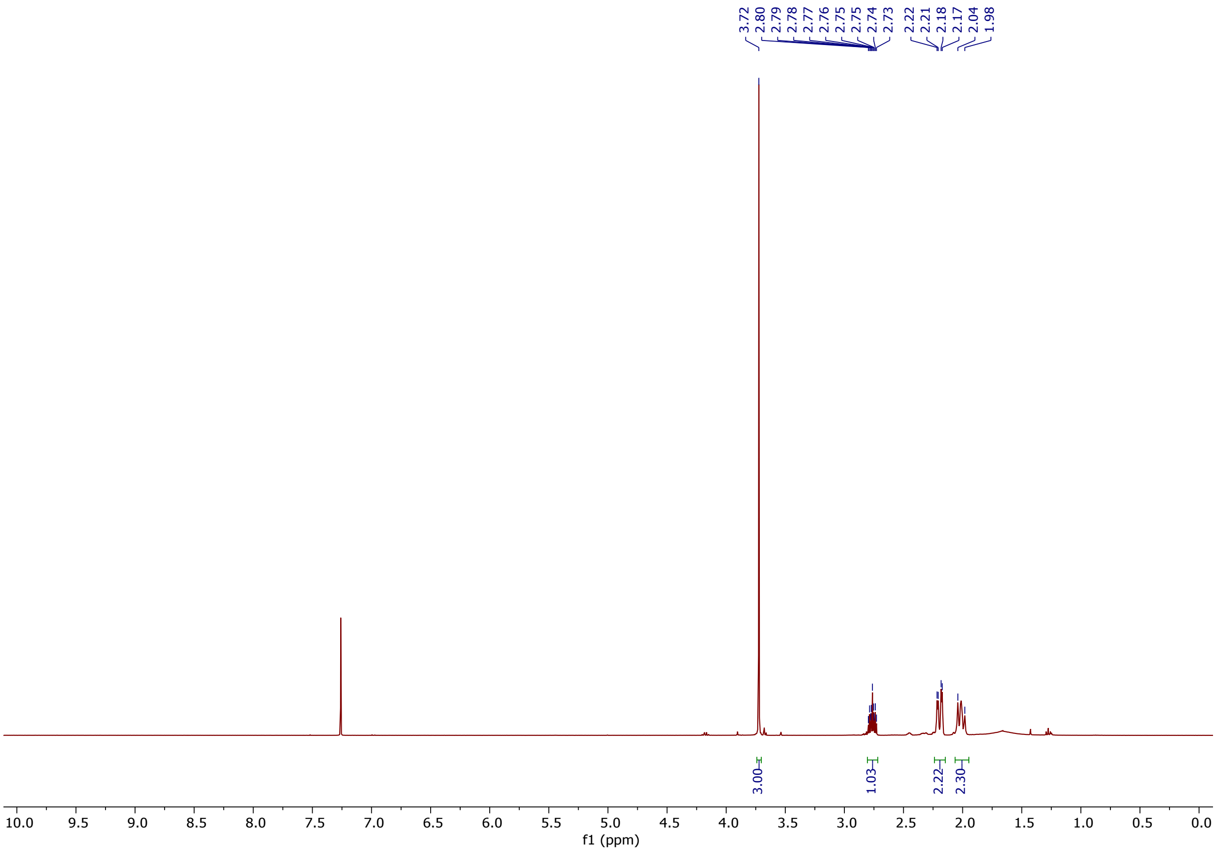

^{13}C NMR spectra of compound **S2** (101 MHz, 298K, $CDCl_3$)

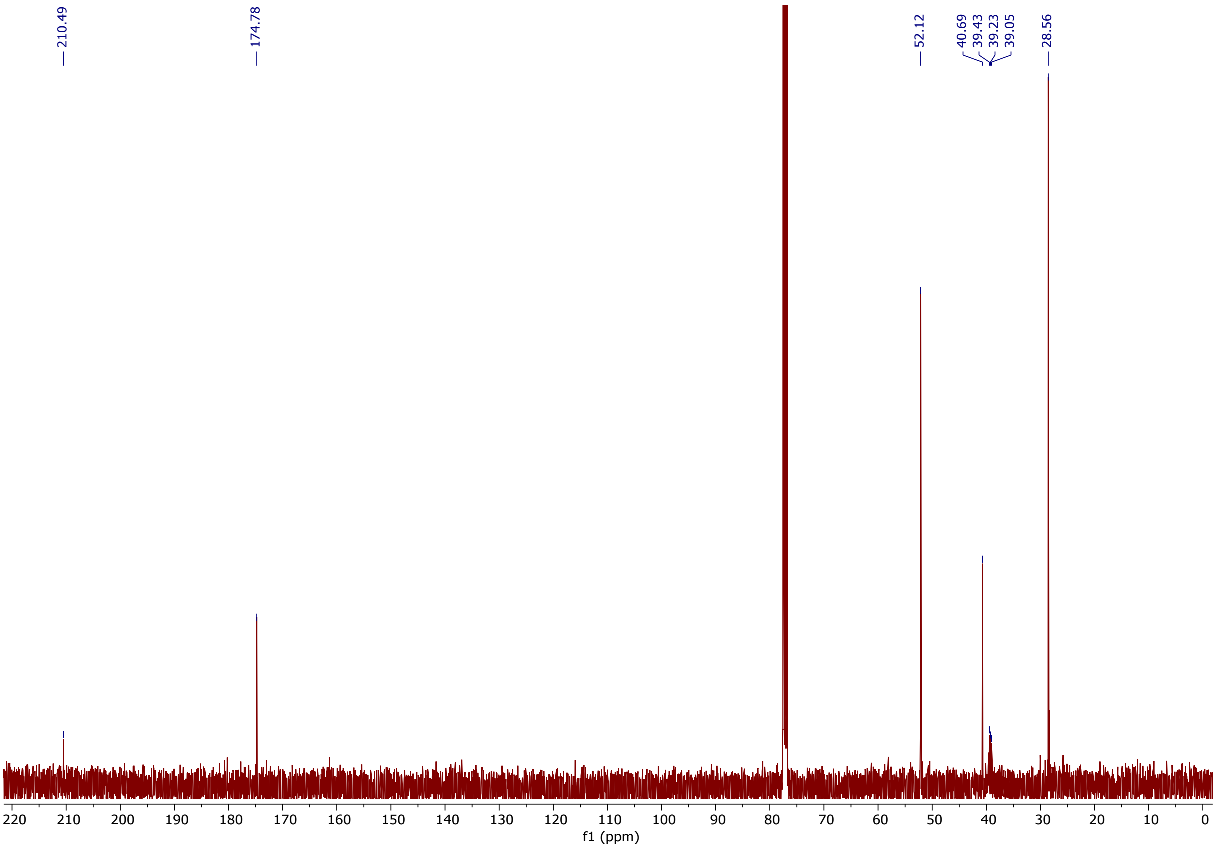

d_3-Triflate **S4**

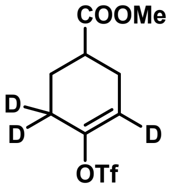

To a dry flask containing *d_4*-ketone **S2** (2.81 g, 17.5 mmol) and *N*-(5-chloropyridin-2-yl)-*N*-(methanesulfonyl)methanesulfonamide (Comin's reagent, 7.57 g, 19.3 mmol) was added dry THF (30 mL) under a nitrogen atmosphere. The mixture was cooled to −78 °C, before 1M sodium hexamethyldisilazane in THF (19.3 mL, 19.3 mmol) was added dropwise. The mixture was stirred at −78 °C for 1 hour, before being warmed to room temperature and stirred overnight. After this, saturated aqueous NH_4Cl (12 mL) was added, before the mixture was evaporated to dryness. Et_2O (60 mL) and 2M aqueous NaOH (30 mL) were added, and the mixture was vigorously stirred for 15 minutes. After this, the organic portion was separated, and the aqueous portion was extracted with Et_2O (x2). The organic portions were combined and sequentially washed with distilled water and then brine, before being dried over Na_2SO_4, filtered, and concentrated *in vacuo*. The crude mixture was subjected to silica gel chromatography (1:0 to 9:1, PET: Et_2O) to afford the title compound as a clear oil (1.027 g, 20%).

^{1}H NMR (400 MHz, 298K, $CDCl_3$) δ 3.71 (s, 3H), 2.60 (dddd, 1H, *J* = 10.5, 7.3, 3.6, 3.6 Hz), 2.48-2.42 (m, 2H), 2.13 (dd, 1H, *J* = 13.4, 3.1 Hz), 1.94-1.91 (m, 1H).

^{13}C NMR (101 MHz, 298K, $CDCl_3$) δ 174.5, 148.4, 116.8 (t, 24.9 Hz), 52.1, 37.7, 26.1, 25.9, 25.8, 25.6, 24.9.

HRMS (ESI-MS) calculated for **S4** $C_9H_9D_3F_3O_5S^+$ [M + H]$^+$: 292.2630; not found.

^{1}H NMR spectra of compound **S4** (400 MHz, 298K, $CDCl_3$)

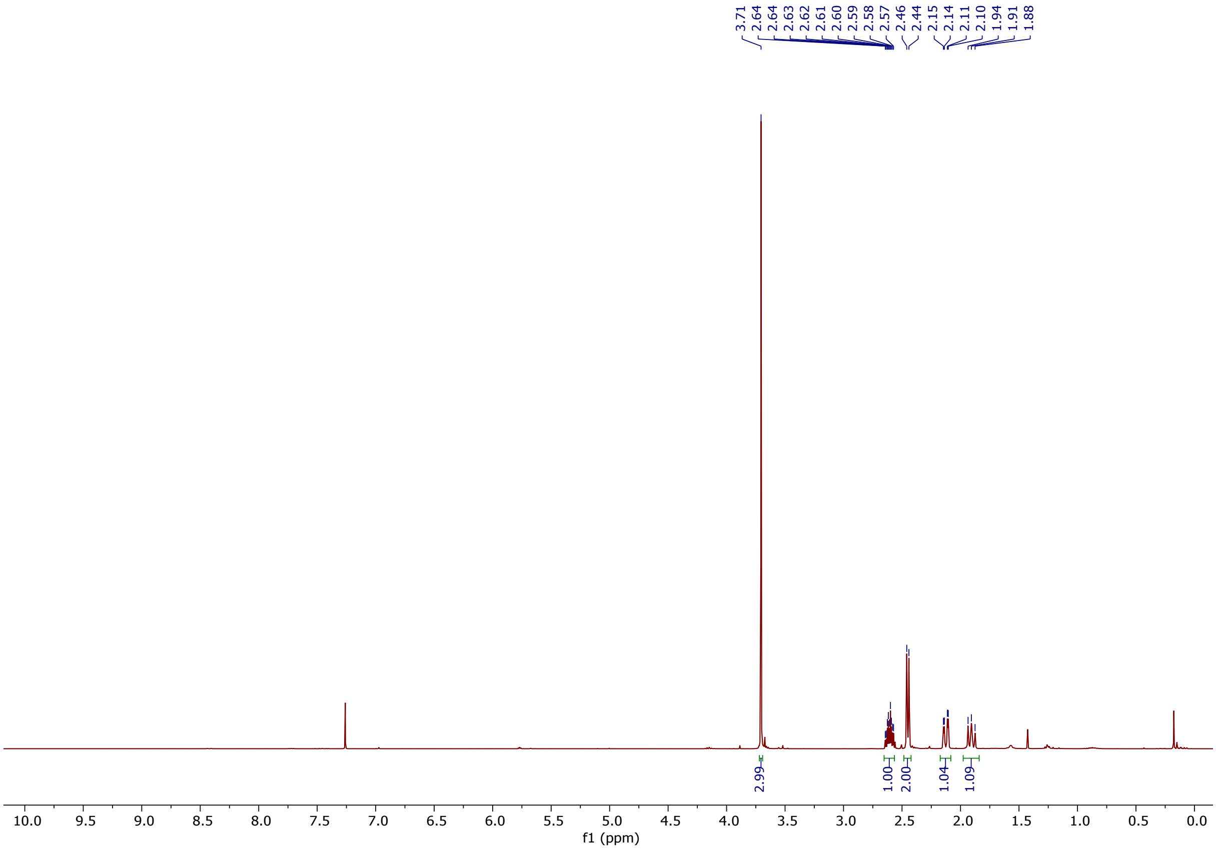

^{13}C NMR spectra of compound **S4** (101 MHz, 298K, $CDCl_3$)

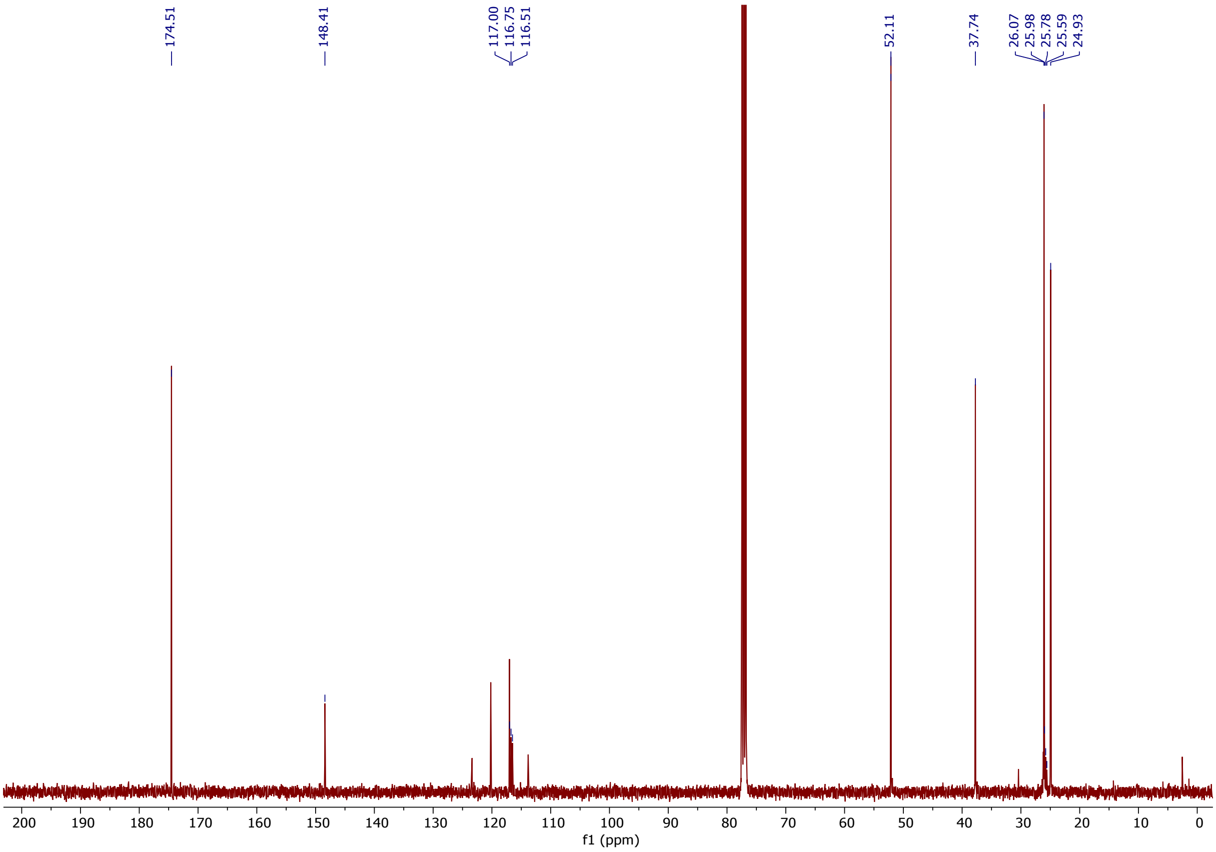

d_3-Diene **S6**

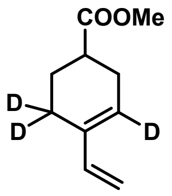

To a dry sealable glass vessel containing d_4-triflate **S4** (1.027 g, 3.526 mmol) lithium chloride (0.450 g, 10.6 mmol), palladium acetate (0.040 g, 0.18 mmol), and (ferrocene-1,1'-diyl)bis(diphenylphosphane) (0.096 g, 0.18 mmol), was added dry THF (15 mL), followed by vinyl tributyl tin (1.12 g, 1.04 mL, 3.53 mmol) under a nitrogen atmosphere. The mixture was pump purged using nitrogen gas three times, before being heated to 100 °C and stirred overnight. At completion, the reaction mixture was filtered through Celite using Et_2O, and dried *in vacuo*. The crude mixture was subjected to silica gel chromatography (0:1 to 1:9, Et_2O: PET, immediately after loading PET (250 mL) was flushed through the column to remove as much leftover tributyltin hydroxide as possible) to provide the title compound (0.330 g, 53%, adjusted for $SnBu_3OH$ contamination) as a clear oil.

^{1}H NMR (400 MHz, 298K, $CDCl_3$) δ 6.35 (dd, 1H, J = 17.6, 10.8 Hz), 5.09-5.05 (m, 1H), 4.95-4.92 (m, 1H), 3.69 (s, 3H), 2.56 (dddd, 1H, J = 11.6, 8.7, 6.8, 3.0 Hz), 2.39-2.37 (m, 2H), 2.09 (dd, 1H, J = 13.2, 2.8 Hz), 1.74-1.68 (m, 1H).

^{13}C NMR (101 MHz, 298K, $CDCl_3$) δ 176.2, 139.4, 135.6, 127.2 (t, J = 24.4 Hz), 110.8, 51.8, 39.4, 28.1, 24.9. CD_2 signal not observed.

HRMS (ESI-MS) calculated for **S6** $C_{10}H_{12}D_3O_2^+$ $[M + H]^+$: 170.1255; not found.

^{1}H NMR spectra of compound **S6** (400 MHz, 298K, $CDCl_3$)

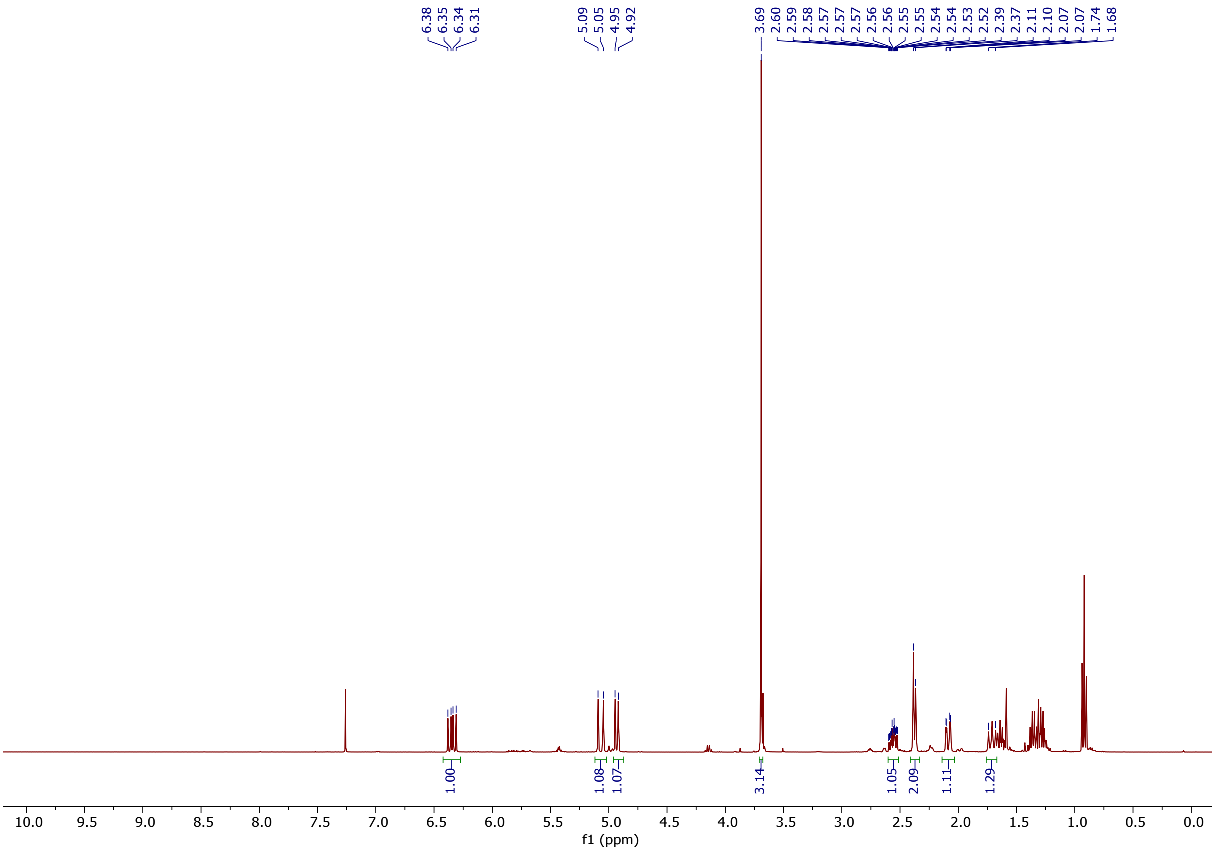

^{13}C NMR spectra of compound **S6** (101 MHz, 298K, $CDCl_3$)

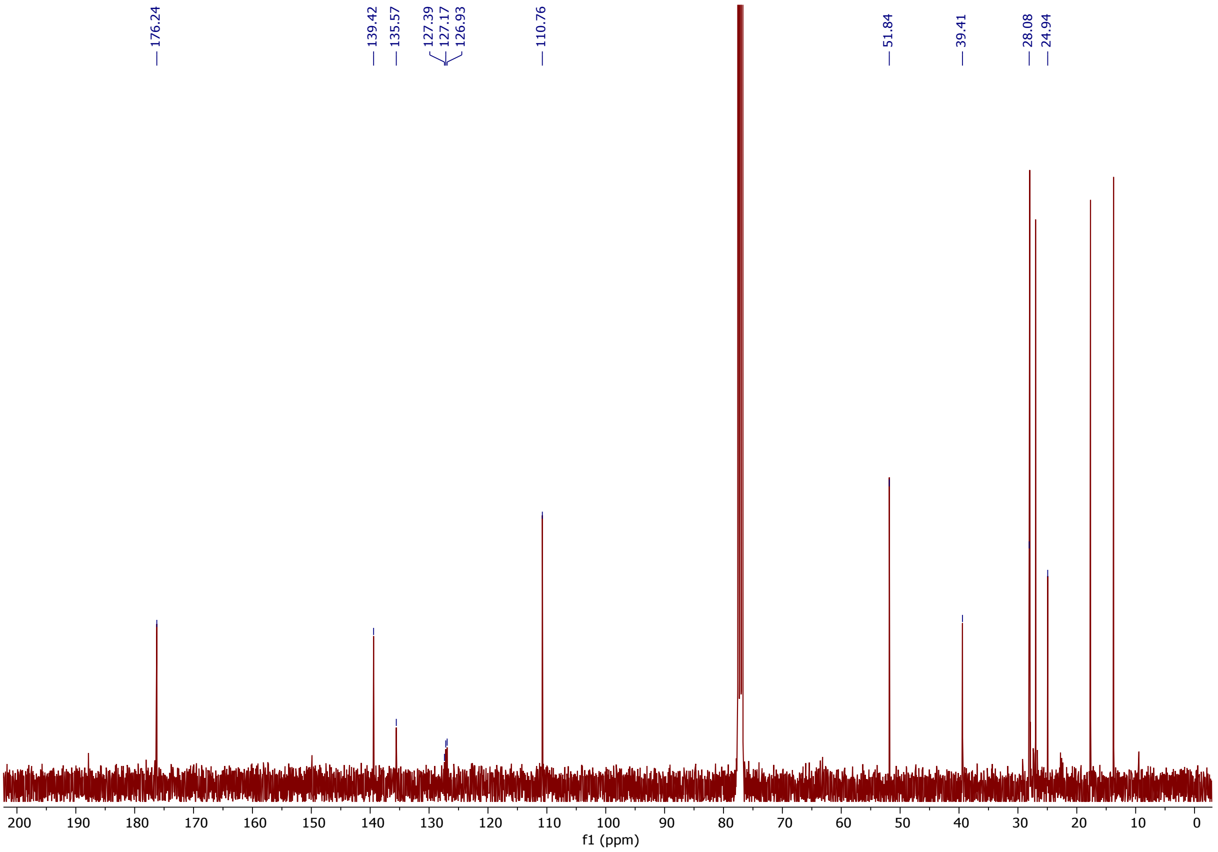

d_2-Alkene **S10**

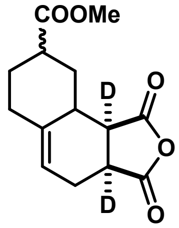

A solution of d_3-diene **S6** (0.108 g, 0.649 mmol) and d_2-maleic anhydride (0.065 g, 0.649 mmol) in toluene (3 mL) was pump purged with nitrogen in a sealable glass vessel. The mixture was heated to 80 °C and stirred overnight. At completion, the solvent was removed *in vacuo*, and the crude residue subjected to silica gel column chromatography (1:1:98 to 30:1:69, EtOAc:AcOH:PET) to afford the title product (0.124 g, 62%) as a mixture of diastereomers.

^{1}H NMR (400 MHz, 298K, $CDCl_3$) δ 5.61-5.44 (m, 1H), 3.69 (s, 3H), 2.96-2.46 (m, 3H), 2.46-2.15 (m, 4H), 2.10-1.49 (m, 3H).

^{13}C NMR (101 MHz, 298K, $CDCl_3$) δ 175.9, 174.9, 174.0, 173.4, 171.8, 171.4, 140.3, 138.2, 117.9, 117.3, 52.01, 51.98, 43.4 (t, J = 21.1 Hz), 42.8, 39.5 (t, J = 21.0 Hz), 37.9, 33.8, 33.1, 31.5, 31.4, 29.8, 28.6, 27.6, 26.0, 23.1, 21.6.

HRMS (ESI-MS) calculated for **S10** $C_{14}H_{15}D_2O_6^-$ $[M + OH]^-$: 283.1156; found: 283.1158.

^{1}H NMR spectra of compound **S10** (400 MHz, 298K, $CDCl_3$)

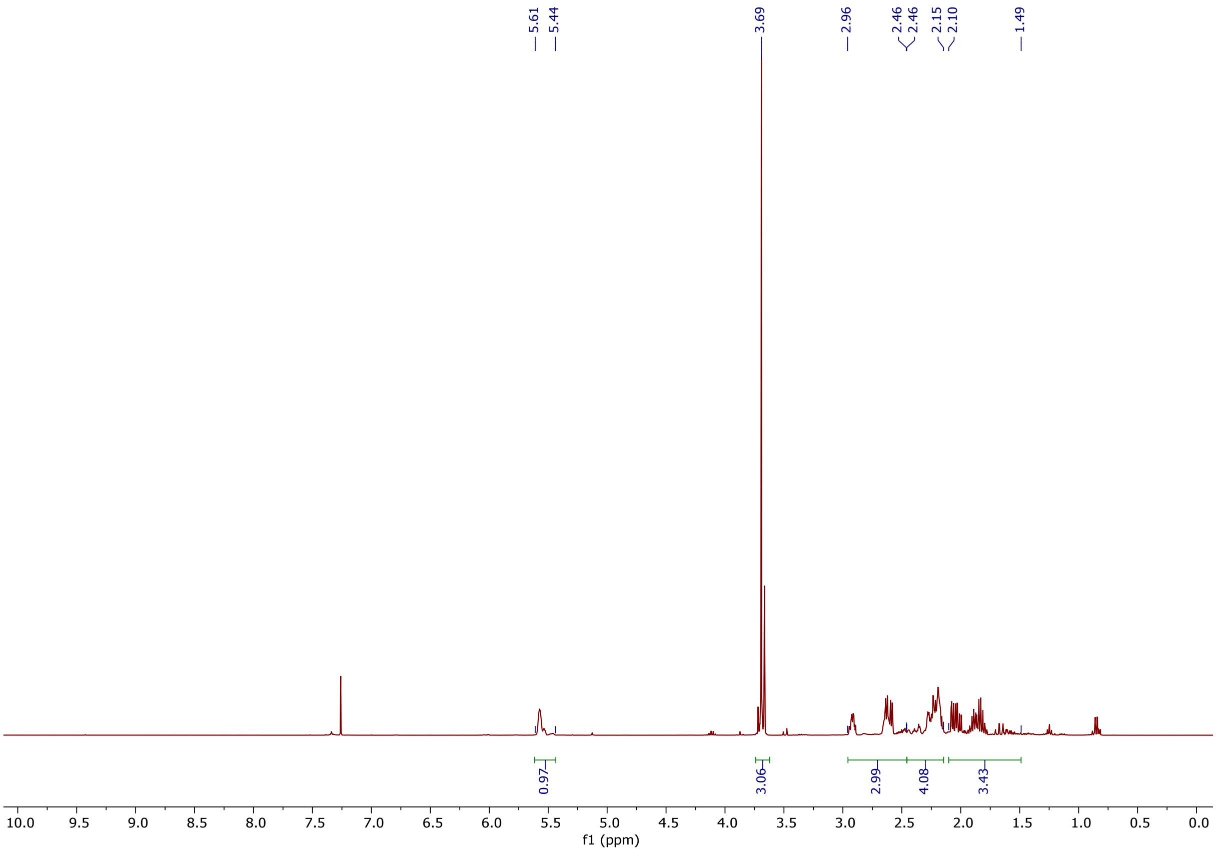

^{13}C NMR spectra of compound **S10** (101 MHz, 298K, $CDCl_3$)

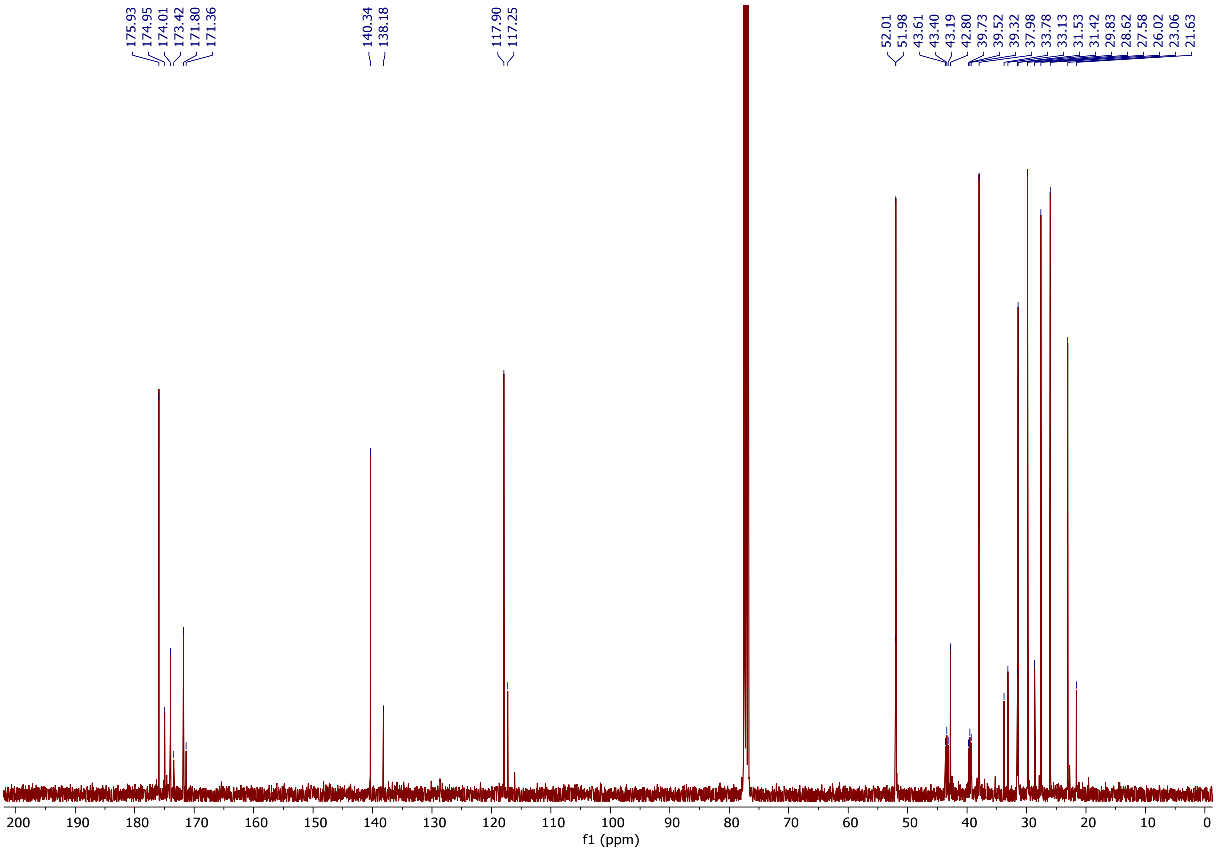

d_3-Alkene **S11**

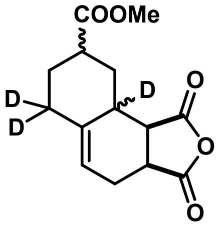

A solution of *d_3*-diene **S6** (0.089 g, 0.52 mmol) and maleic anhydride (0.052 g, 0.52 mmol) in toluene (3 mL) was pump purged with nitrogen in a sealable glass vessel. The mixture was heated to 80 °C and stirred overnight. At completion, the solvent was removed *in vacuo*, and the crude residue subjected to silica gel column chromatography (1:1:98 to 30:1:69, EtOAc:AcOH:PET) to afford the title product (0.116 g, 82%) as a mixture of diastereomers (major diastereomers: all minor and trace diastereomers, 3:1).

Mixture ^{1}H NMR (400 MHz, 298K, $CDCl_3$) δ 5.56-5.43 (m, 1H), 3.70-3.63 (m, 3H), 3.43-3.18 (m, 2H), 2.92-2.70 (m, 1H), 2.64-2.54 (m, 1H), 2.36-2.10 (m, 2H), 2.04-1.92 (m, 1H), 1.88-1.49 (m, 2H).

Major diastereomer ^{1}H NMR (400 MHz, 298K, $CDCl_3$) δ 5.56 (dd, 1H, *J* = 5.8, 3.3 Hz), 3.67 (s, 3H), 3.42-3.30 (m, 2H), 2.89 (dddd, 1H, *J* = 7.4, 6.1, 5.5, 2.7 Hz), 2.59 (ddd, 1H, *J* = 16.8, 5.9, 2.5 Hz), 2.25 (ddd, 1H, *J* = 16.7, 7.2, 3.2 Hz), 2.21 (dd, 1H, *J* = 14.0, 2.7 Hz) 2.00 (dd, 1H, *J* = 13.7, 6.1 Hz), 1.86 (abx, 1H, *J* = 13.6, 5.5 Hz), 1.79 (abx, 1H, *J* = 13.6, 7.4 Hz).

Mixture ^{13}C NMR (101 MHz, 298K, $CDCl_3$) δ 175.9, 174.9, 174.0, 173.5, 171.8, 171.4, 140.2, 138.0, 117.9, 117.2, 51.94, 51.92, 43.7, 42.8, 42.6, 39.8, 38.7, 37.9, 33.4 (t, *J* = 19.7 Hz), 31.4, 31.0 (t, *J* = 20.0 Hz), 29.1 (t, *J* = 19.3 Hz), 28.4, 27.5, 25.9, 23.1, 21.7.

Major diastereomer ^{13}C NMR (101 MHz, 298K, $CDCl_3$) δ 175.9, 174.0, 171.8, 140.2, 117.9, 51.9, 43.7, 39.8, 37.9, 31.0 (t, *J* = 20.0 Hz), 29.1 (t, *J* = 19.3 Hz), 27.5, 25.8, 23.1.

HRMS (ESI-MS) calculated for **S11** $C_{14}H_{14}D_3O_6^-$ $[M + OH]^-$: 284.1219; found: 284.1222.

^{1}H NMR spectra of compound **S11** integrated and peak picked for major isomer (400 MHz, 298K, $CDCl_3$)

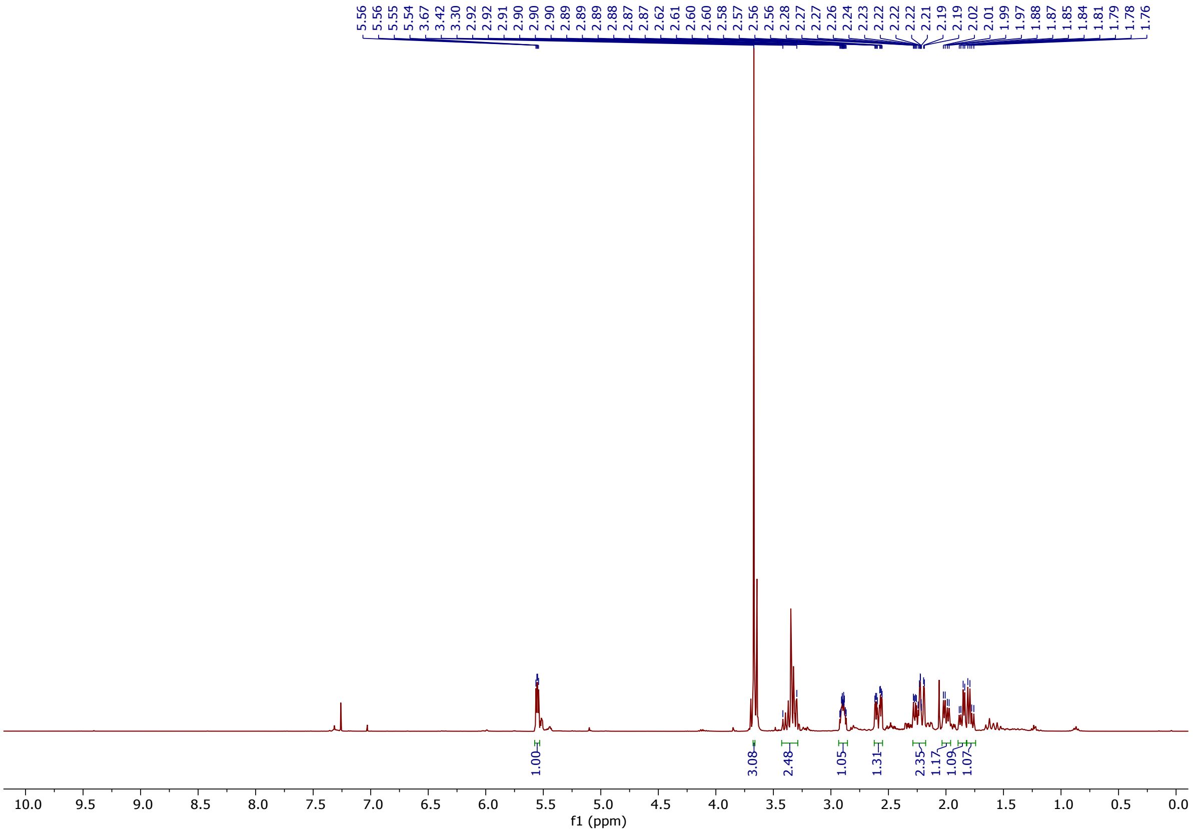

^{1}H NMR spectra of compound **S11** integrated and peak picked for all isomers (400 MHz, 298K, $CDCl_3$)

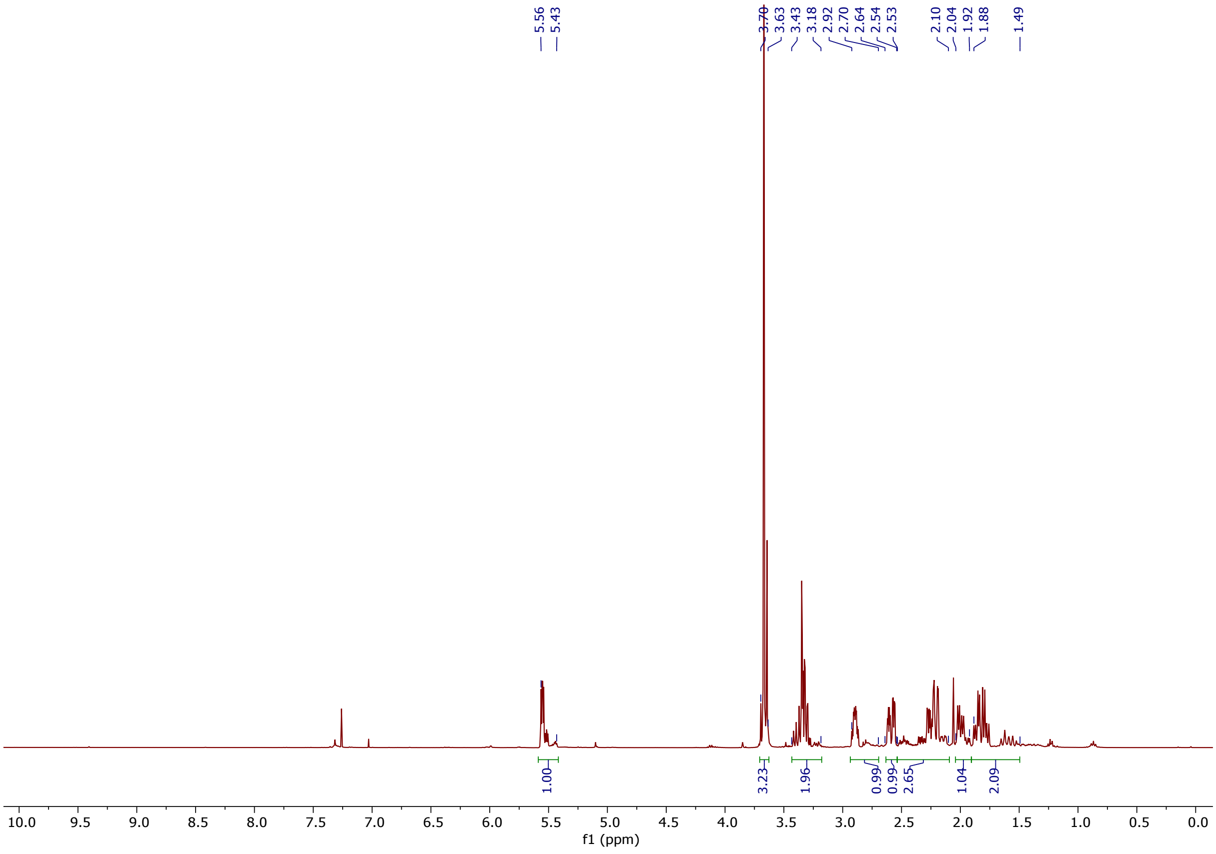

^{13}C NMR spectra of compound **S11** (101 MHz, 298K, $CDCl_3$)

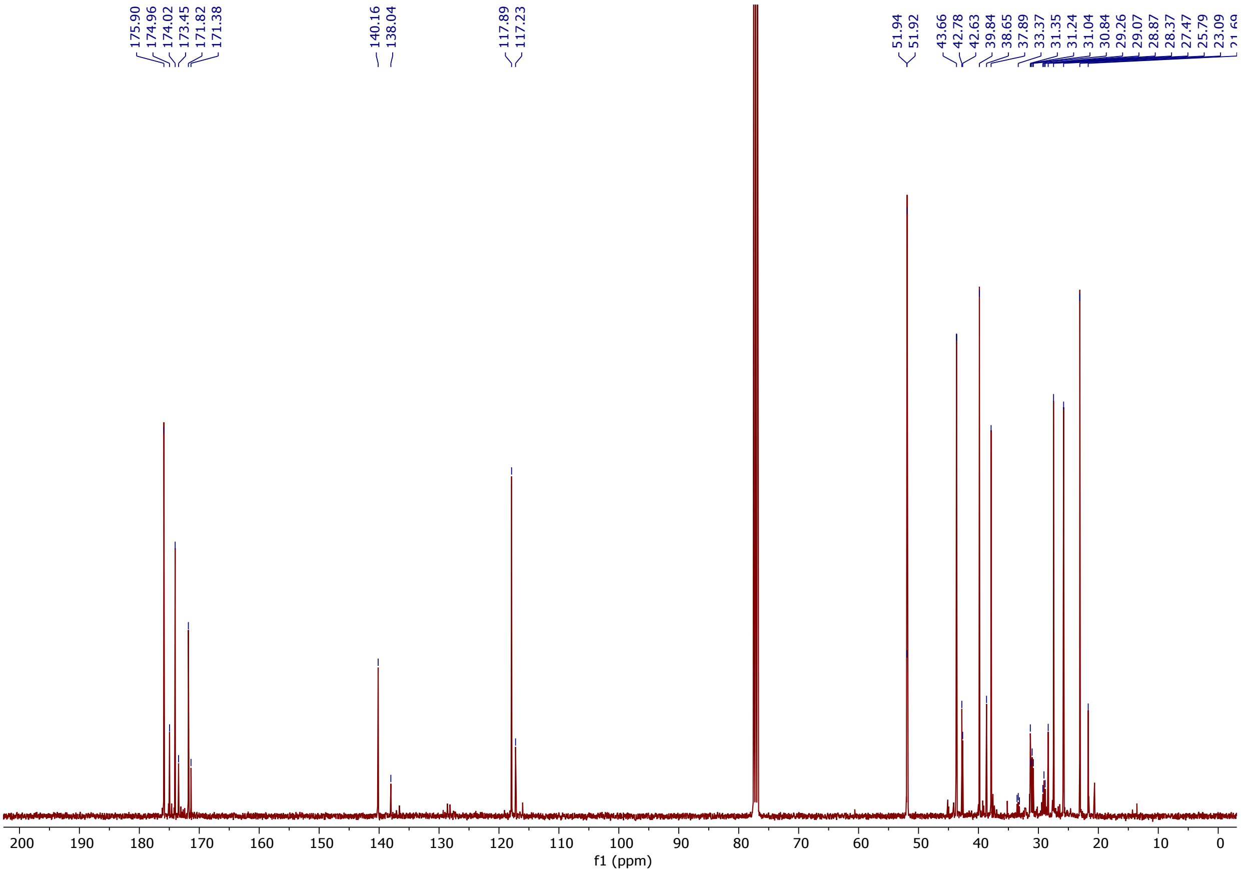

d_5-Alkene **S12**

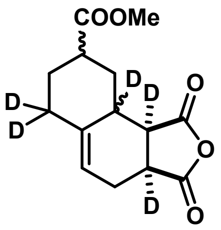

A solution of *d_3*-diene **S6** (0.208 g, 1.23 mmol) and *d_2*-maleic anhydride (0.123 g, 1.23 mmol) in toluene (3 mL) was pump purged with nitrogen in a sealable glass vessel. The mixture was heated to 80 °C and stirred overnight. At completion, the solvent was removed *in vacuo*, and the crude residue subjected to silica gel column chromatography (1:1:98 to 30:1:69, EtOAc:AcOH:PET) to afford the title product (0.234 g, 70%) as a mixture of diastereomers (major diastereomers: all minor and trace diastereomers, 3:1).

Mixture ^{1}H NMR (400 MHz, 298K, $CDCl_3$) δ 5.56-5.43 (m, 1H), 3.70-3.65 (m, 3H), 2.92-2.69 (m, 1H), 2.64-2.54 (m, 1H), 2.35-2.10 (m, 2H), 2.04-1.92 (m, 1H), 1.88-1.49 (m, 2H).

Major diastereomer ^{1}H NMR (400 MHz, 298K, $CDCl_3$) δ 5.56 (dd, 1H, *J* = 5.8, 3.3 Hz), 3.67 (s, 3H), 2.89 (dddd, 1H, *J* = 7.4, 6.1, 5.5, 2.7 Hz), 2.59 (dd, 1H, *J* = 16.8, 5.9, Hz), 2.25 (dd, 1H, *J* = 13.8, 3.3 Hz), 2.21 (dd, 1H, *J* = 13.7, 2.8 Hz) 2.00 (dd, 1H, *J* = 13.6, 6.1 Hz), 1.86 (abx, 1H, *J* = 13.7, 5.6 Hz), 1.79 (abx, 1H, *J* = 13.7, 7.3 Hz).

Mixture ^{13}C NMR (101 MHz, 298K, $CDCl_3$) δ 175.9, 175.0, 174.1, 173.5, 171.8, 171.4, 140.1, 138.0, 117.9, 117.2, 52.02, 51.97, 51.94, 51.8, 43.6, 43.2 (t, *J* = 20.9 Hz), 42.7, 39.4 (t, *J* = 21.0 Hz), 37.9, 35.1, 33.2 (t, *J* = 20.9 Hz), 31.5, 31.4, 30.9 (t, *J* = 20.3 Hz), 29.1 (t, *J* = 19.5 Hz), 28.4, 27.5, 25.8, 25.8, 22.9, 21.6, 21.5.

Major diastereomer ^{13}C NMR (101 MHz, 298K, $CDCl_3$) δ 175.9, 174.1, 171.8, 140.1, 117.9, 52.02, 43.2 (t, *J* = 20.9 Hz), 39.4 (t, *J* = 21.0 Hz), 37.9, 31.0 (t, *J* = 20.0 Hz), 29.1 (t, *J* = 19.3 Hz), 27.5, 25.8, 23.9.

HRMS (ESI-MS) calculated for **S12** $C_{14}H_{12}D_5O_6^-$ $[M + OH]^-$: 286.1344; found: 286.1344.

¹H NMR spectra of compound **S12** integrated and peak picked for major isomer (400 MHz, 298K, $CDCl_3$)

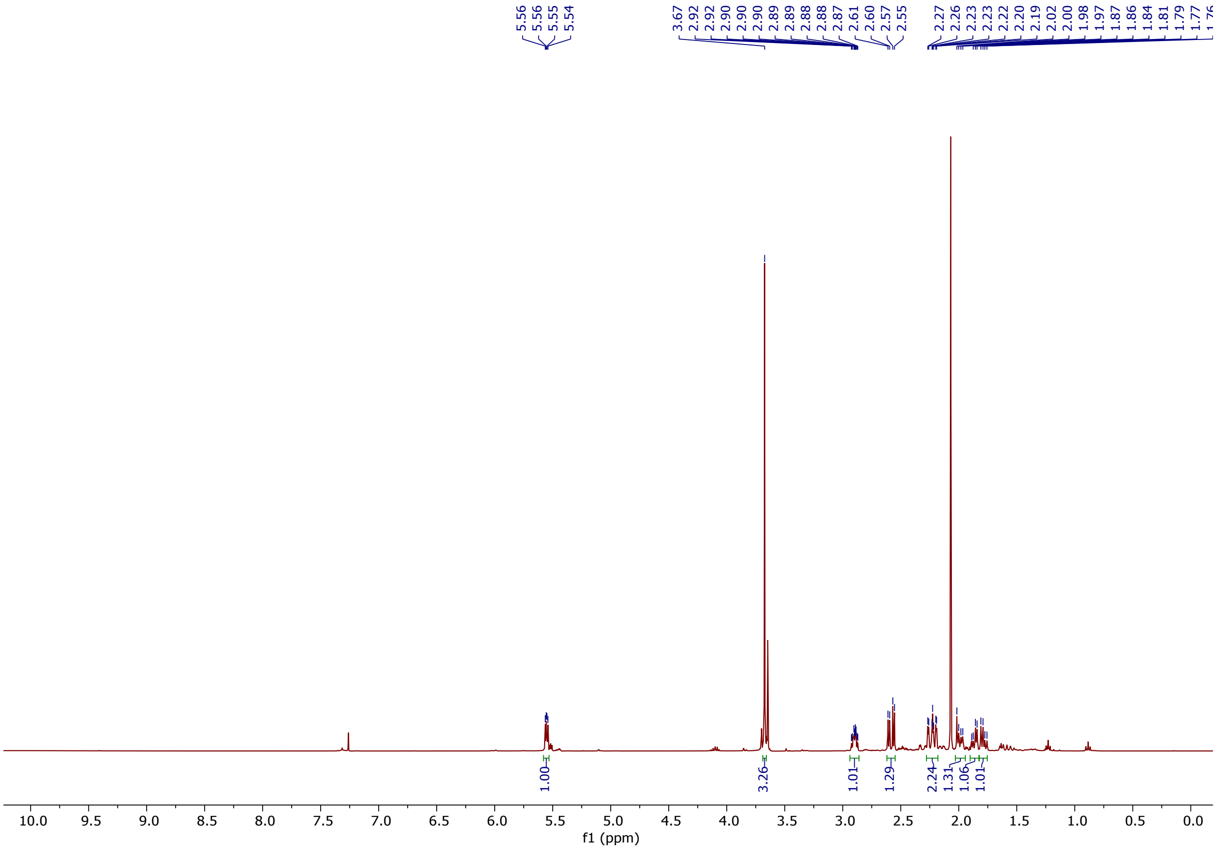

¹H NMR spectra of compound **S12** integrated and peak picked for all isomers (400 MHz, 298K, $CDCl_3$)

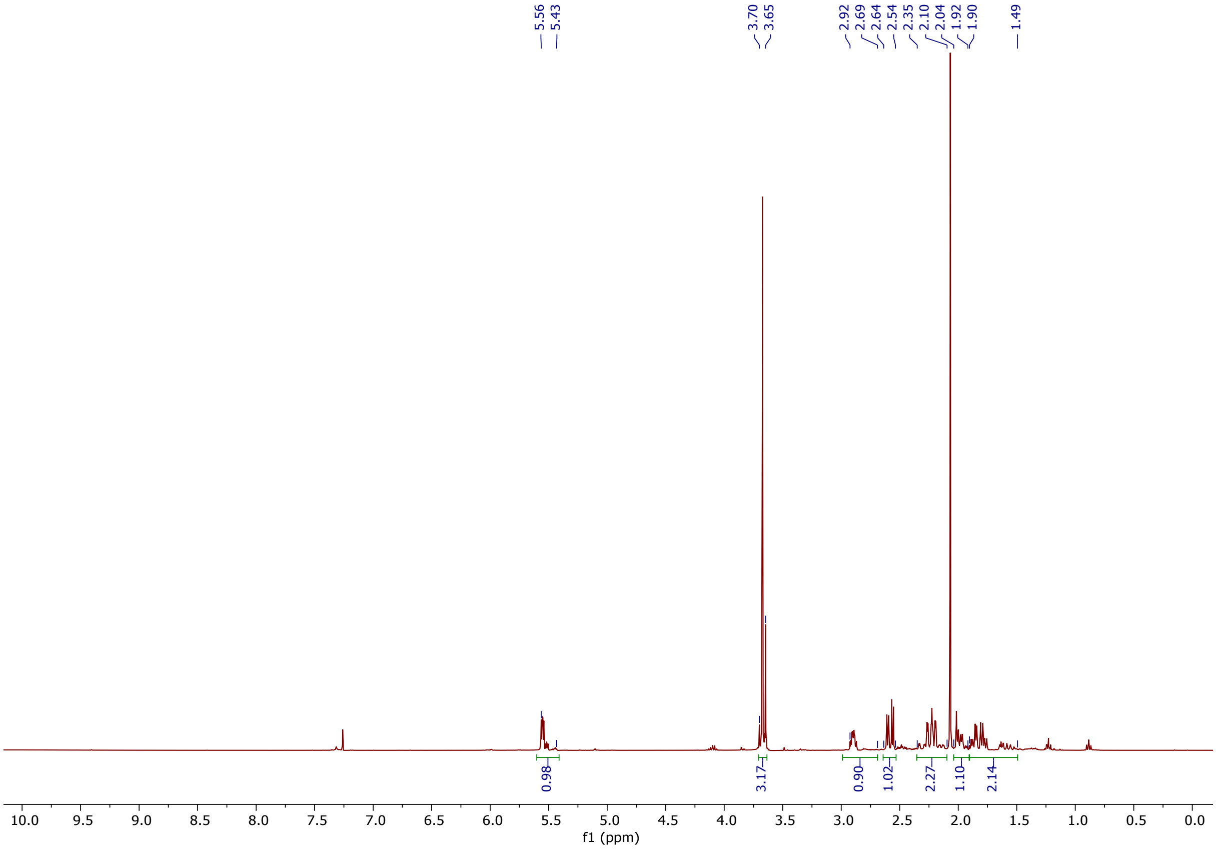

^{13}C NMR spectra of compound **S12** (101 MHz, 298K, $CDCl_3$)

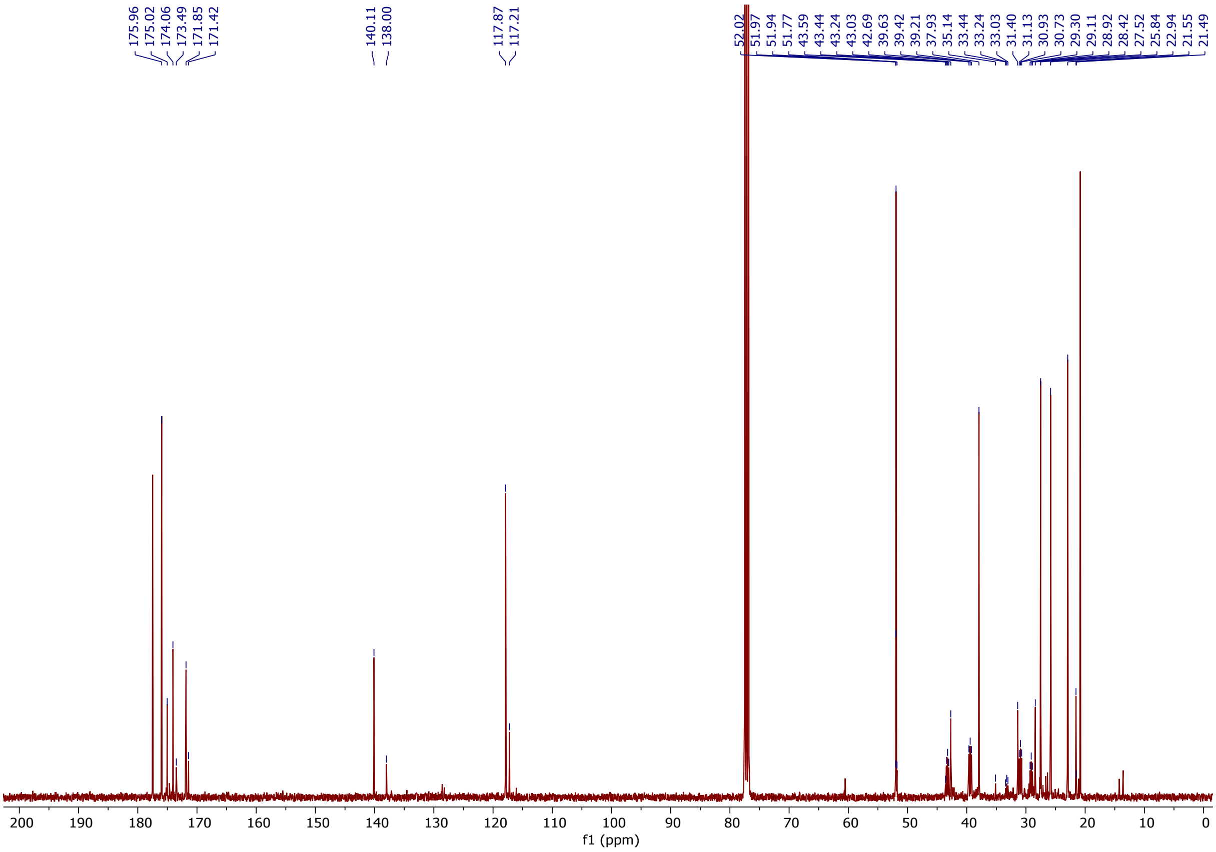

d_2-Triacid ***d_2*-1**

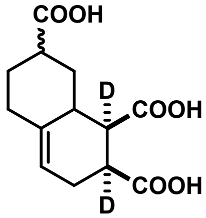

To a solution of d_2-alkene **S10** (0.092 g, 0.38 mmol) in methanol (1 mL) was added 25% aqueous NaOH solution (9 mL). The mixture was stirred overnight at room temperature. At completion, the mixture was washed with diethyl ether (x2), before being acidified to pH 2, and extracted with ethyl acetate (x3). The resulting solution was concentrated in vacuo, before the remaining liquid was frozen and freeze-dried to afford the title compound as an off-white powder (0.091 g, 97%).

^{1}H NMR (500 MHz, 298K, MeOD) δ 5.59-5.39 (m, 1H), 2.85-2.37 (m, 3H), 2.36-2.09 (m, 4H), 1.56-1.26 (m, 2H).

^{13}C NMR picked for major isomer (126 MHz, 298K, MeOD) δ 178.3, 177.6, 176.5, 136.8, 120.3, 45.6 (t, *J* = 19.9 Hz), 42.2 (17.9 Hz), 40.3, 35.9, 32.0, 31.6, 28.5, 25.7.

^{13}C NMR picked above arbitrary threshold (126 MHz, 298K, MeOD) δ 179.2, 179.1, 178.7, 178.6, 178.4, 178.3, 178.0, 177.6, 177.5, 177.0, 176.5, 140.7, 139.8, 139.4, 136.8, 120.3, 119.0, 118.6, 117.9, 45.6 (t, *J* = 19.9 Hz), 42.2, 42.1, 42.0, 40.9, 40.3, 40.2, 38.8, 38.2, 38.1, 36.7, 36.1, 35.9, 35.5, 34.9, 34.7, 33.8, 33.5, 32.2, 32.0, 31.9, 31.6, 30.5, 30.2, 29.2, 29.0, 28.5, 25.7.

HRMS (ESI-MS) calculated for ***d_2*-1** $C_{13}H_{14}D_2O_6^-$ [M - H]$^-$: 269.1000; found: 269.0997.

^{1}H NMR spectra of compound ***d*$_2$-1** (400 MHz, 298K, $CDCl_3$)

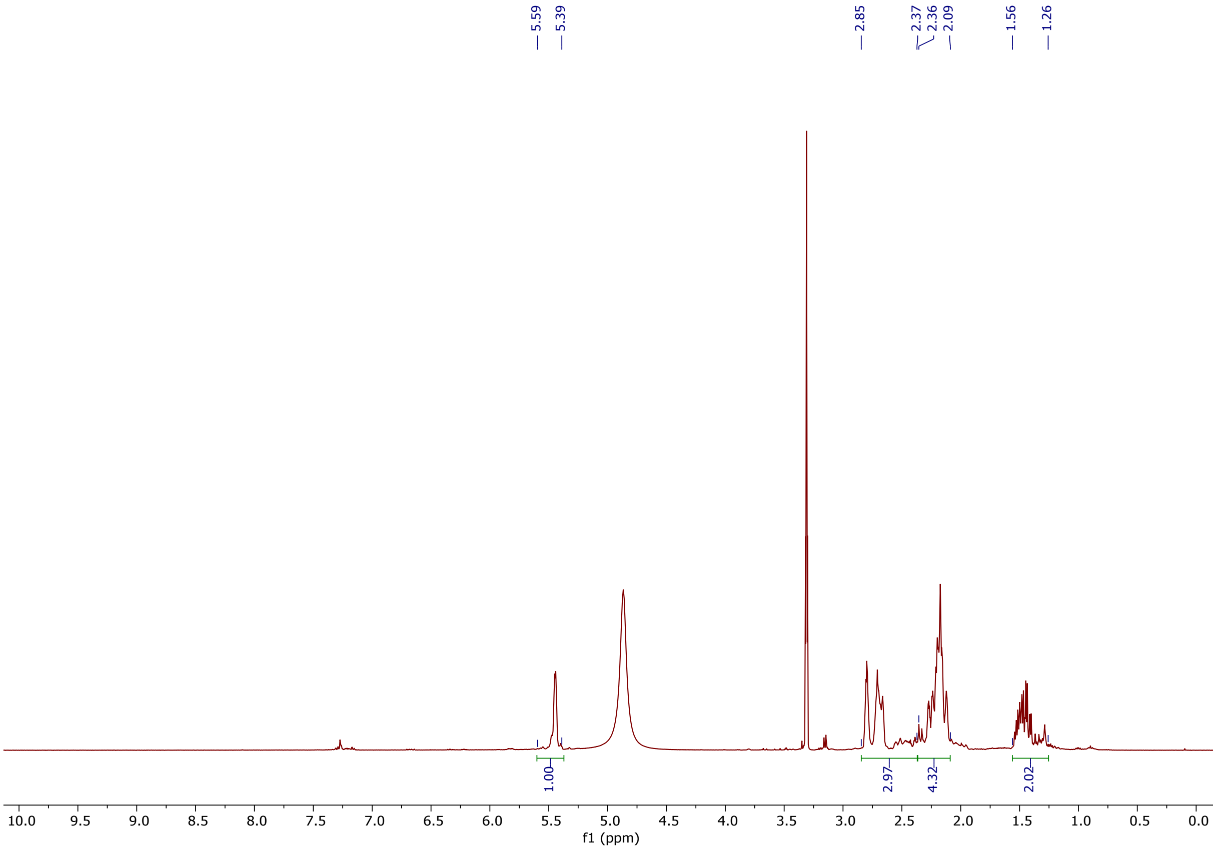

^{13}C NMR spectra of compound ***d*$_2$-1**, peaks picked for major isomer (126 MHz, 298K, $CDCl_3$)

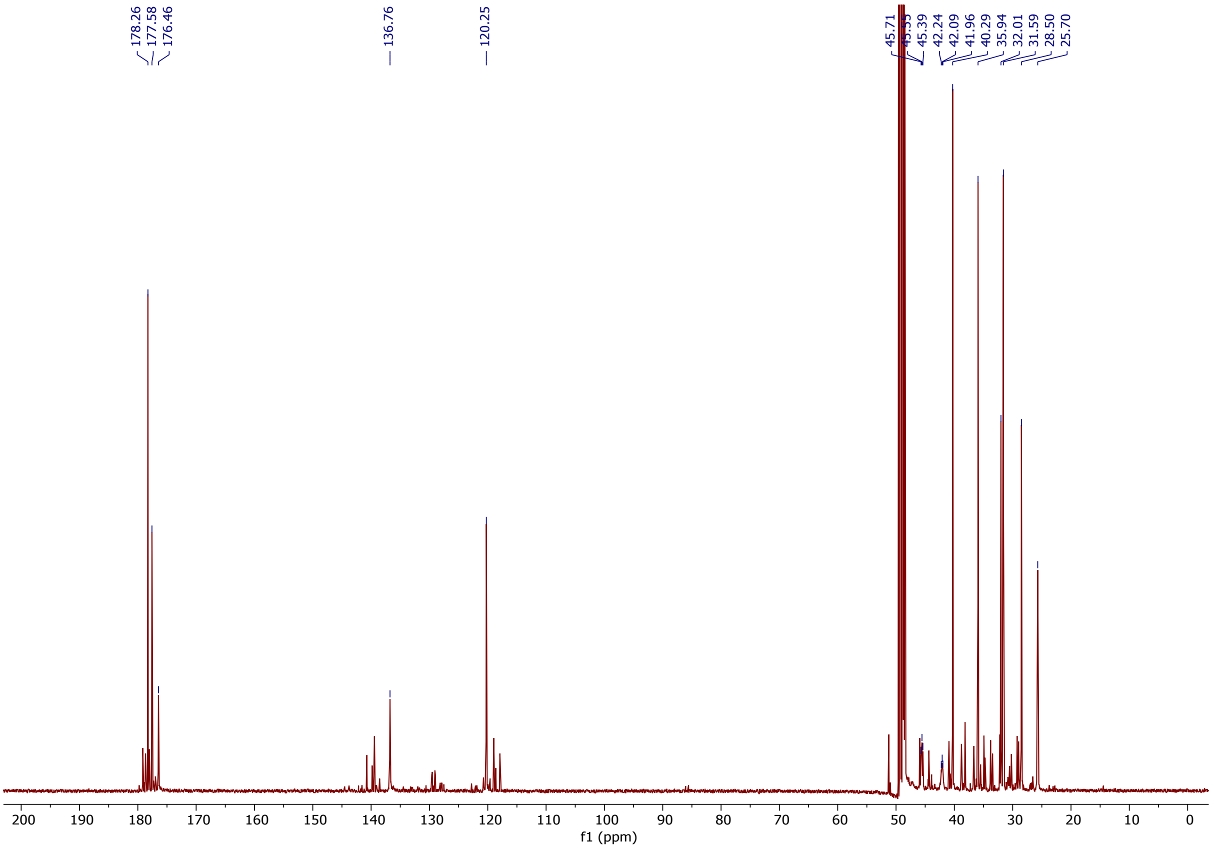

^{13}C NMR spectra of compound ***d₂*-1**, peaks picked above an arbitrary threshold (126 MHz, 298K, $CDCl_3$)

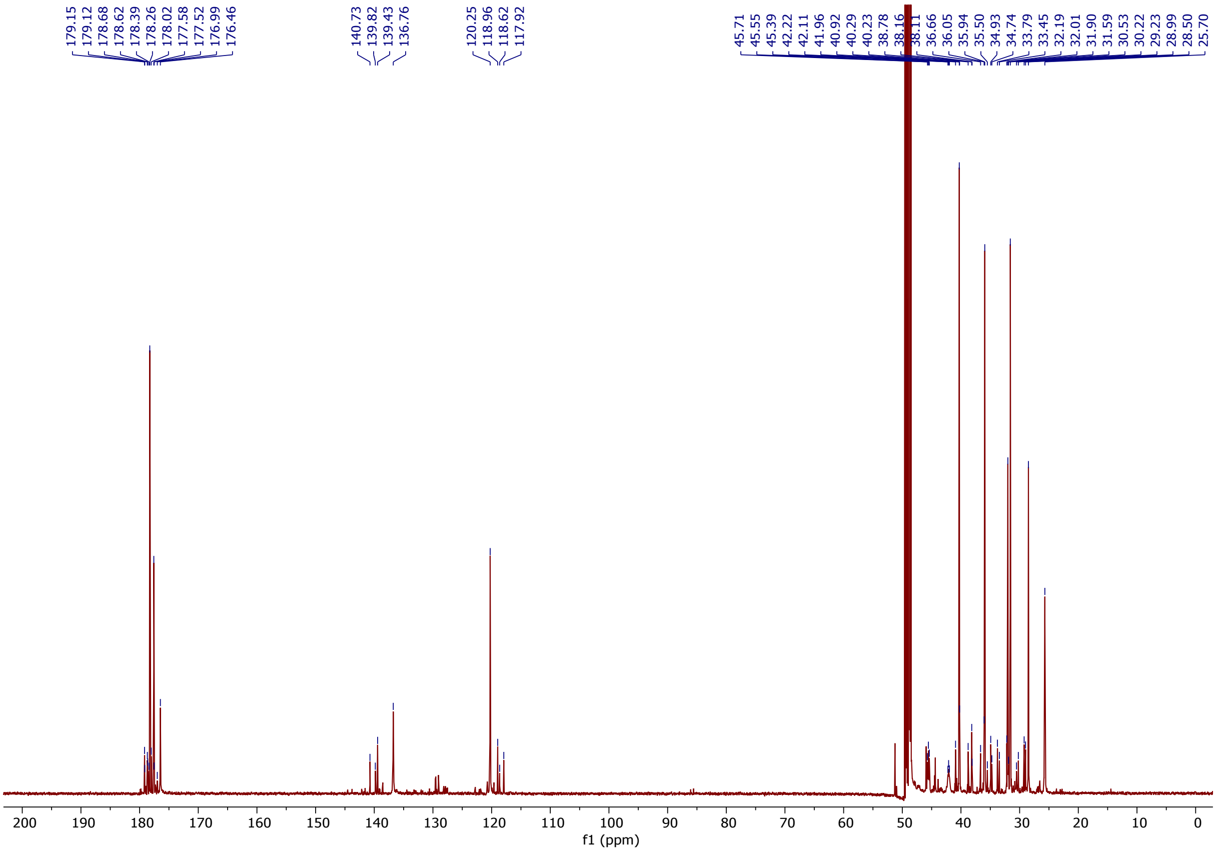

d_3-Triacid ***d_3*-1**

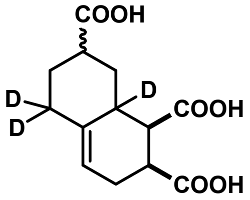

To a solution of *d_3*-alkene **S6** (0.070 g, 0.26 mmol) in methanol (0.5 mL) was added 25% aqueous NaOH solution (2 mL). The mixture was stirred overnight at room temperature. At completion, the mixture was washed with diethyl ether (x2), before being acidified to pH 2, and extracted with ethyl acetate (x3). The resulting solution was concentrated in vacuo, before the remaining liquid was frozen and freeze-dried to afford the title compound as an off-white powder (0.063 g, 88%).

Mixture ^{1}H NMR (400 MHz, 298K, $CDCl_3$) δ 5.51-5.39 (m, 1H), 3.19-2.32 (m, 4H), 2.31-1.94 (m, 3H), 1.54-1.24 (m, 2H).

Major diastereomer ^{1}H NMR (400 MHz, 298K, $CDCl_3$) δ 5.44 (dd, 1H, *J* = 5.0, 2.6 Hz), 3.15 (d, 1H, *J* = 3.6 Hz), 2.81-2.75 (m, 2H), 2.67 (ddd, 1H, *J* = 17.0, 10.4, 2.9 Hz), 2.25-2.10 (m, 3H), 1.50-1.44 (m, 2H).

Mixture ^{13}C NMR (126 MHz, 298K, $CDCl_3$) δ 179.2, 178.8, 178.4, 178.3, 178.1, 177.9, 140.6, 139.3, 138.5, 137.1, 120.1, 119.6, 119.0, 118.1, 49.5, 46.4, 44.5, 43.8, 42.2, 40.9, 40.4, 40.2, 36.0, 34.8 (t, *J* = 18.0 Hz), 33.7, 31.6, 29.3, 28.8, 28.5, 26.2.

Major diastereomer ^{13}C NMR (126 MHz, 298K, $CDCl_3$) δ 178.3, 177.91, 177.89, 137.1, 120.1, 46.4, 42.2, 40.4, 34.8 (t, *J* = 18.0 Hz), 31.6, 31.5, (C-*CD_2*-C), 28.5, 26.2.

HRMS (ESI-MS) calculated for ***d_3*-1** $C_{13}H_{13}D_3O_6^-$ [M - H]$^-$: 270.1062; found: 270.1062.

^{1}H NMR spectra of compound ***d$_3$*-1** integrated and peak picked for major isomer (400 MHz, 298K, $CDCl_3$)

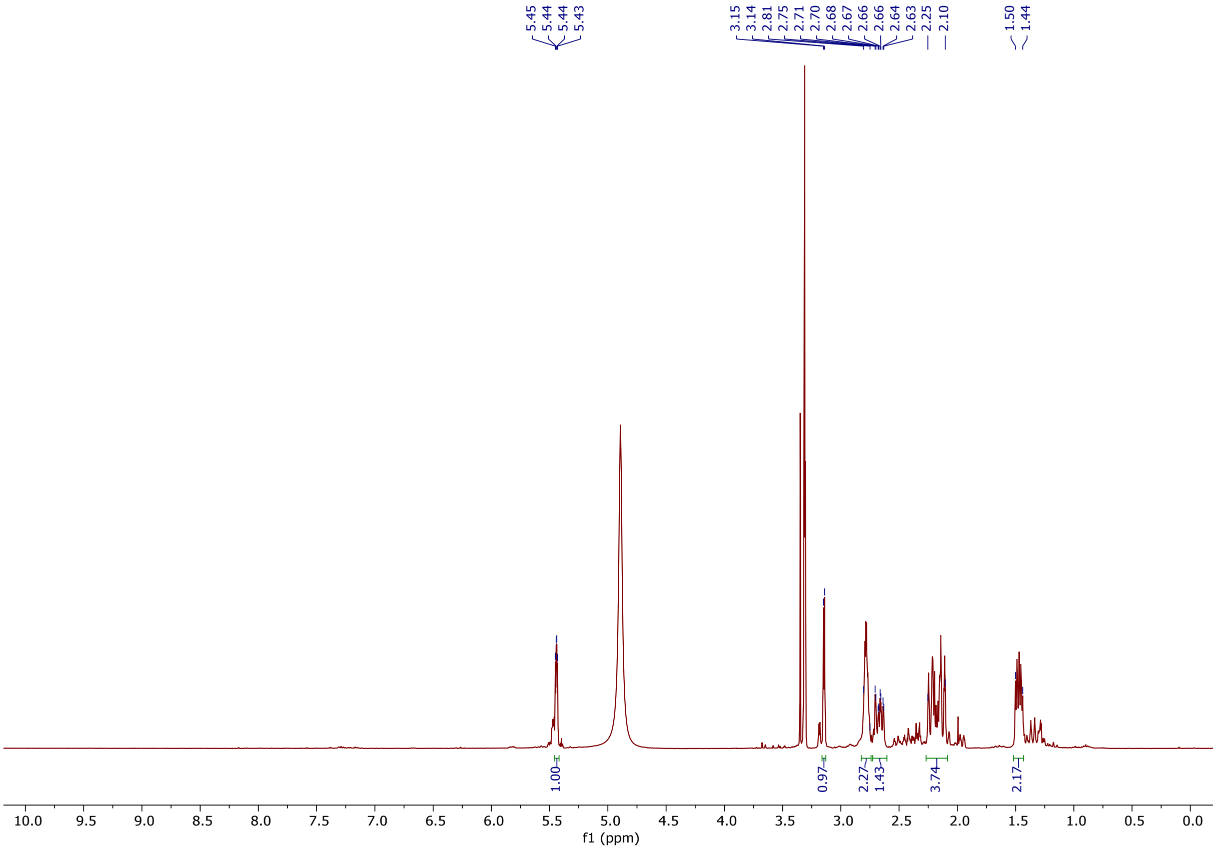

^{1}H NMR spectra of compound ***d$_3$*-1** integrated and peak picked for all isomers (400 MHz, 298K, $CDCl_3$)

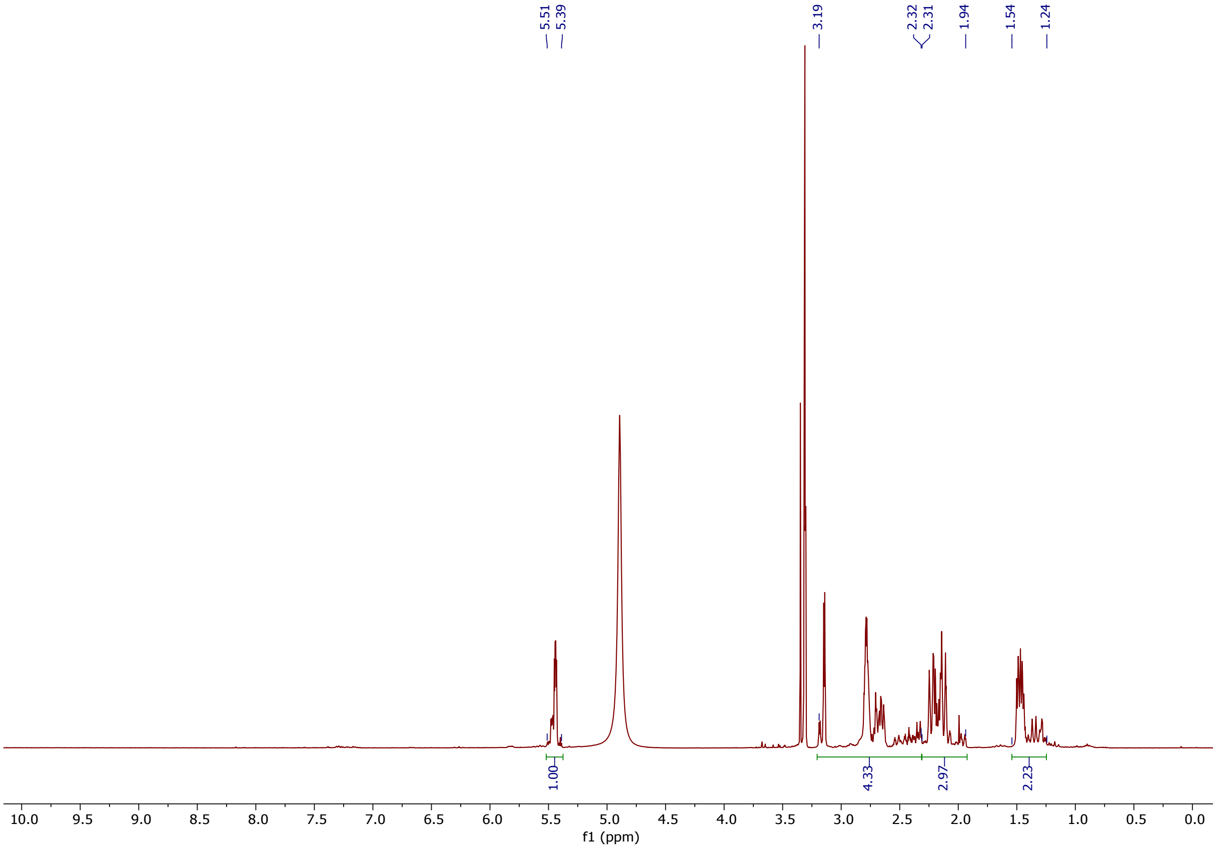

^{13}C NMR spectra of compound ***d*$_3$-1**, peaks picked for major isomer (126 MHz, 298K, $CDCl_3$)

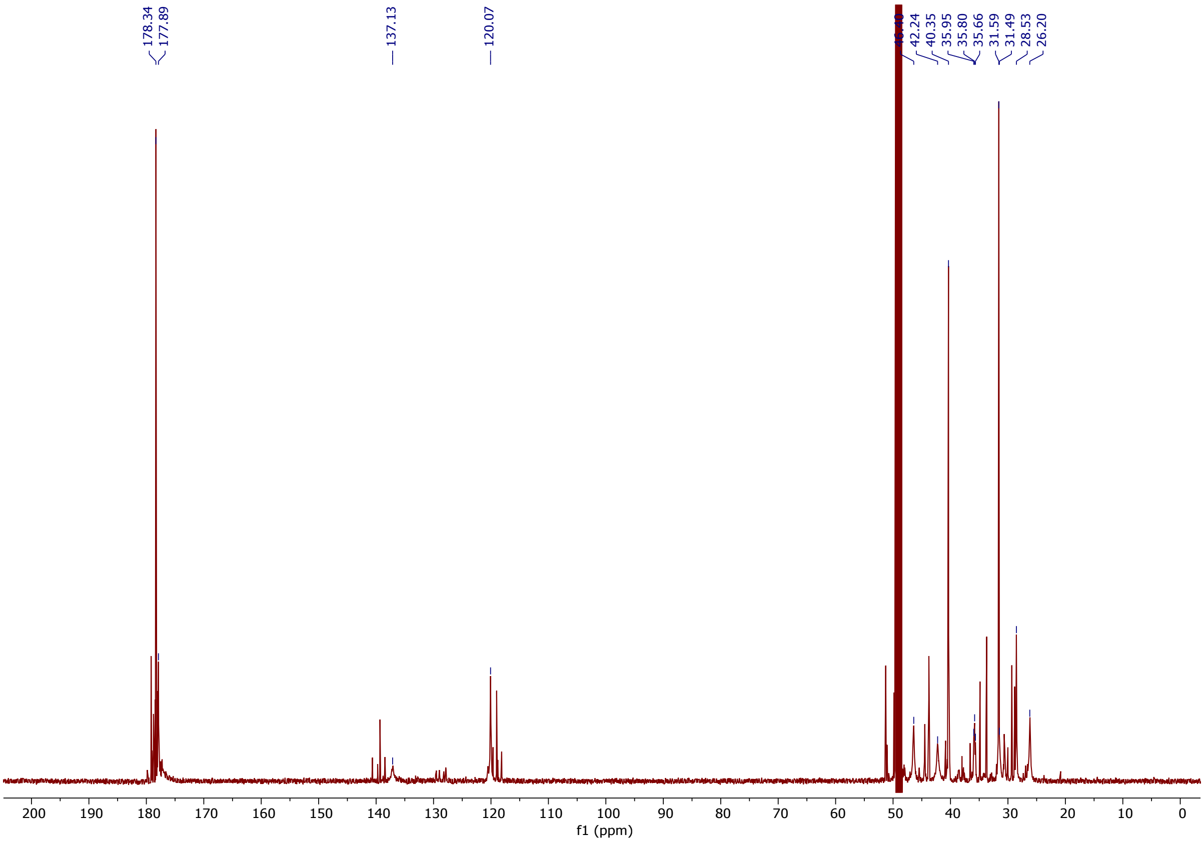

^{13}C NMR spectra of compound ***d*$_3$-1**, peaks picked above an arbitrary threshold (126 MHz, 298K, $CDCl_3$)

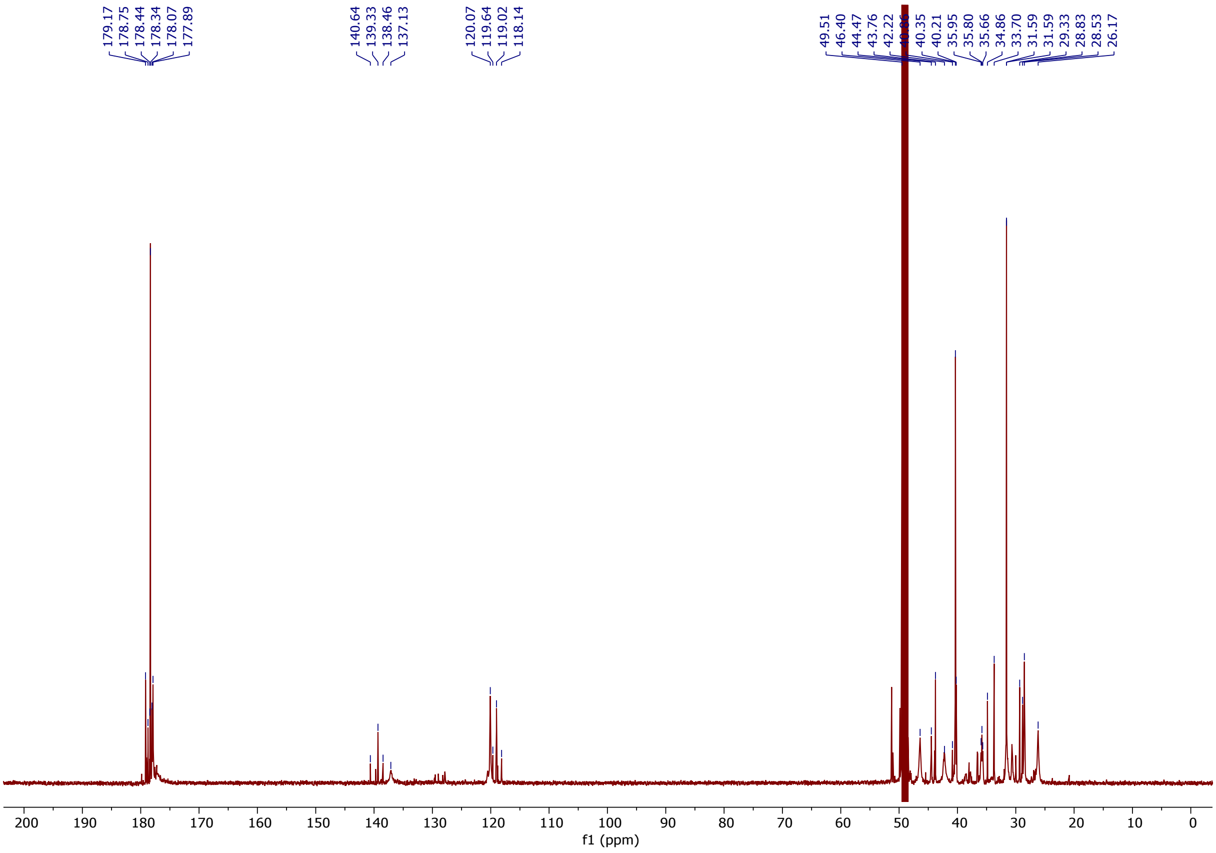

d_5-triacid ***d_5*-1**

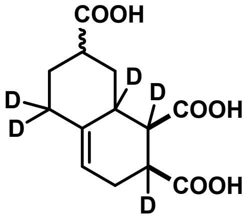

To a solution of *d_3*-alkene **S6** (0.205 g, 0.769 mmol) in methanol (1 mL) was added 25% aqueous NaOH solution (9 mL). The mixture was stirred overnight at room temperature. At completion, the mixture was washed with diethyl ether (x2), before being acidified to pH 2, and extracted with ethyl acetate (x3). The resulting solution was concentrated in vacuo, before the remaining liquid was frozen and freeze-dried to afford the title compound as an off-white powder (0.183 g, 87%). A portion of this material (71 mg) was subjected to preparative HPLC twice (first run: 0.1:10:89.9 to 0.1:20:79.9 HCOOH:MeCN:H_2O, 25 mins, second run of two overlapping peaks isolated from run: 0.1:20:79.9 to 0.1:30:69.9 HCOOH:MeCN:H_2O, 25 mins) to afford two individual isomers (***d_5*-1a**: 2.2 mg, ***d_5*-1b**: 1.3 mg).

***d_5*-mix** ^{1}H NMR (400 MHz, 298K, $CDCl_3$) δ 5.51-5.39 (m, 1H), 2.84-2.38 (m, 2H), 2.37-1.90 (m, 3H), 1.50-1.15 (m, 2H).

***d_5*-1a** ^{1}H NMR (400 MHz, 298K, $CDCl_3$) δ 5.45 (dd, 1H, *J* = 4.9, 2.6 Hz), 2.80 (dddd, 1H, *J* = 4.9, 4.9, 2.8, 2.8 Hz), 2.68 (dd, 1H, *J* = 17.4, 2.1 Hz), 2.25 (ddd, 1H, *J* = 13.1, 1.9, 1.9 Hz), 2.18 (dd, 1H, *J* = 17.3, 5.6 Hz), 2.12 (ddd, 1H, *J* = 13.3, 2.3, 2.3 Hz), 1.48 (dd, 1H, *J* = 13.2, 4.9 Hz), 1.43 (dd, 1H, *J* = 13.1, 4.8 Hz).

***d_5*-1b** ^{1}H NMR (400 MHz, 298K, $CDCl_3$) δ 5.46 (dd, 1H, *J* = 3.8, 3.8 Hz), 2.70-2.60 (m, 2H), 2.47-2.37 (m, 1H), 2.15 (dd, 1H, *J* = 17.4, 3.6 Hz), 2.07-2.03 (m, 1H), 1.98-1.94 (m, 1H), 1.46-1.33 (m, 2H).

***d_5*-mix** ^{13}C NMR (101 MHz, 298K, $CDCl_3$) δ 179.2, 179.1, 178.9, 178.7, 178.6, 178.4, 178.3, 178.0, 177.53, 177.46, 176.40, 176.3, 140.7, 139.3, 136.6, 136.0, 120.8, 120.3, 119.0, 117.9, 45.8, 45.7, 45.6, 45.4, 45.2, 44.4, 44.3, 43.9, 42.1, 40.9, 40.24, 40.20, 38.1, 36.7, 36.6, 35.6, 35.5, 35.3, 34.9, 33.7, 31.6, 31.52, 31.5, 30.7, 30.3, 30.1, 29.2, 28.8, 28.4, 28.3, 25.8, 25.7.

***d_5*-1a** ^{13}C NMR (101 MHz, 298K, $CDCl_3$) δ 178.3, 177.5, 176.4, 136.6, 120.3, 45.4 (t, *J* = 20.3 Hz), 41.2 (t, *J* = 18.3 Hz), 40.2, 35.5 (t, *J* = 18.6 Hz), 31.5, 31.3 (C-*CD_2*-C), 28.3, 25.7.

HRMS (ESI-MS) calculated for ***d_5*-1** $C_{13}H_{11}D_5O_6^-$ [M - H]$^-$: 272.1188; found: 272.1186.

^{1}H NMR spectra of compound ***d$_5$*-1** integrated and peak picked for all isomers (500 MHz, 298K, $CDCl_3$)

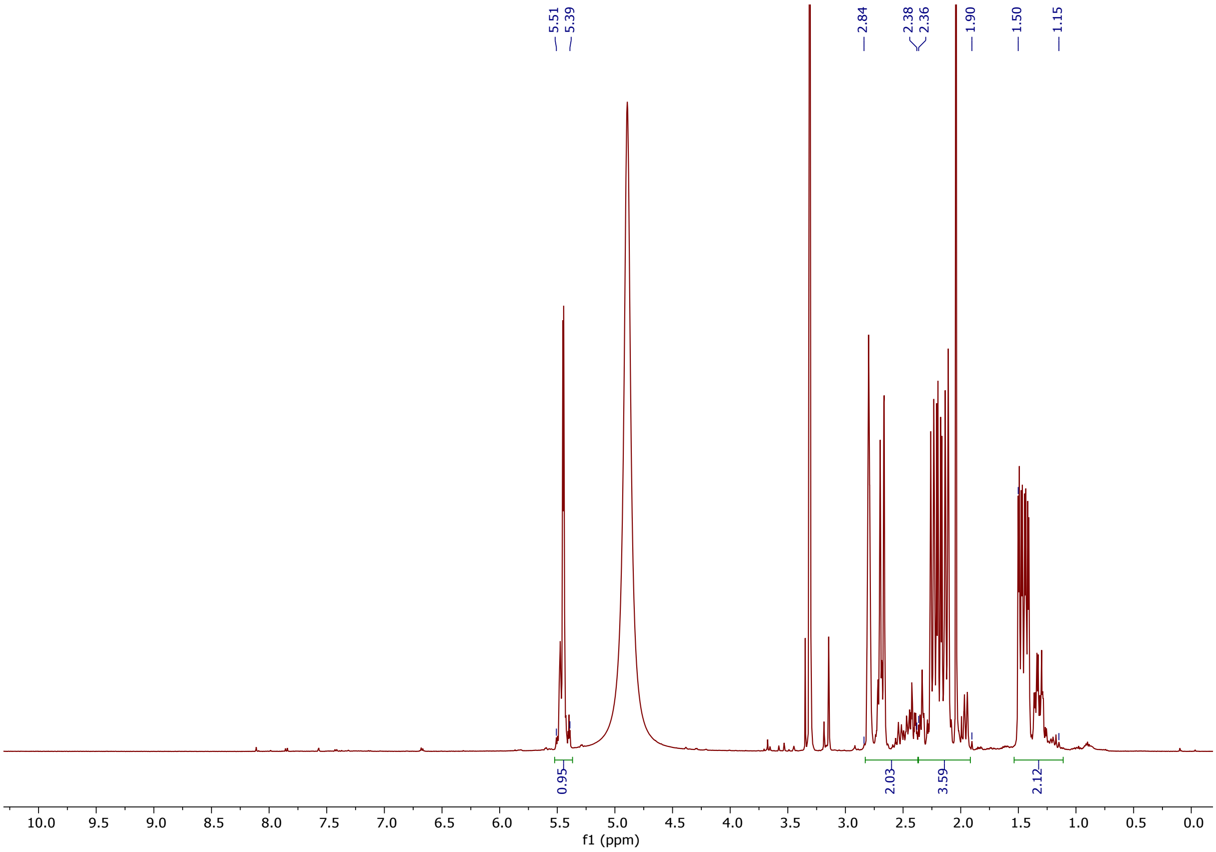

^{1}H NMR spectra of compound ***d$_5$*-1**, integrated and peak picked for major isomer ***d$_5$*-1a** (500 MHz, 298K, $CDCl_3$)

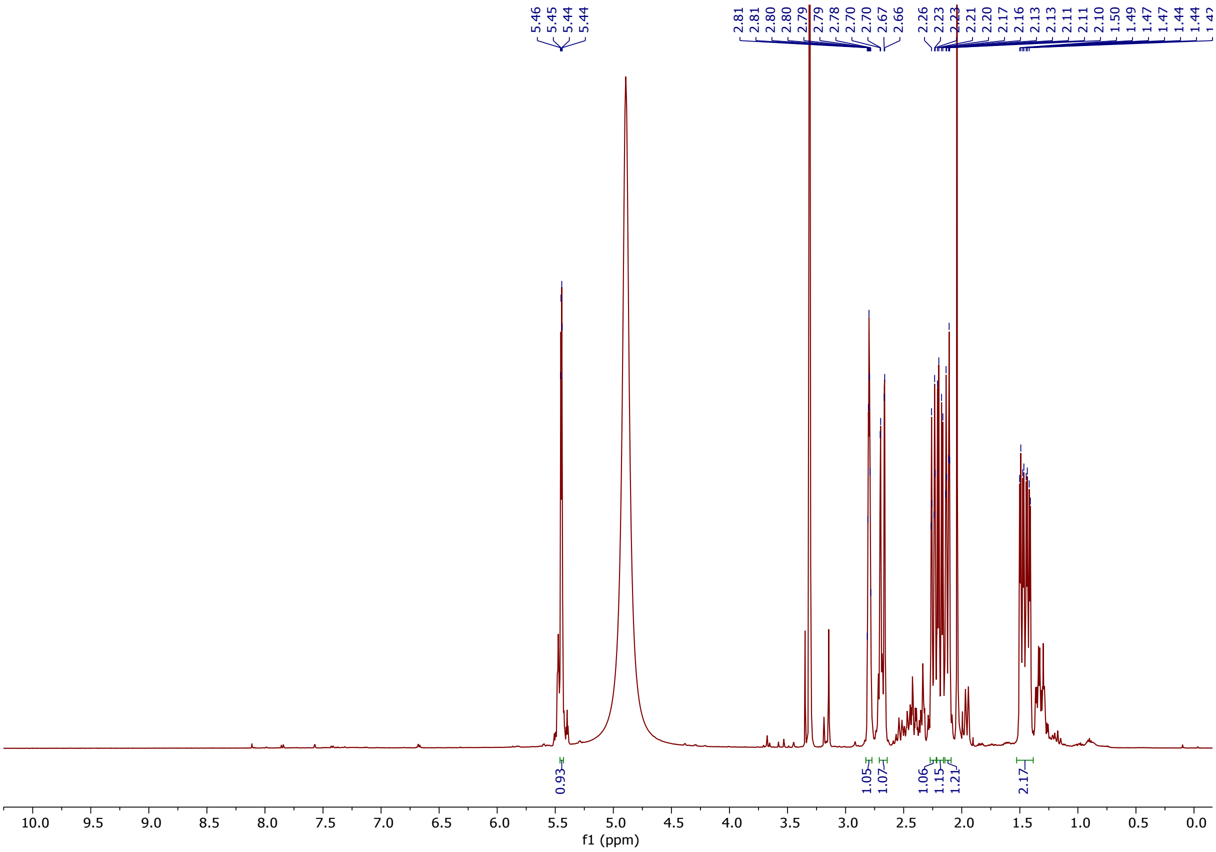

^{1}H NMR spectra of compound ***d$_5$*-1a** (400 MHz, 298K, $CDCl_3$)

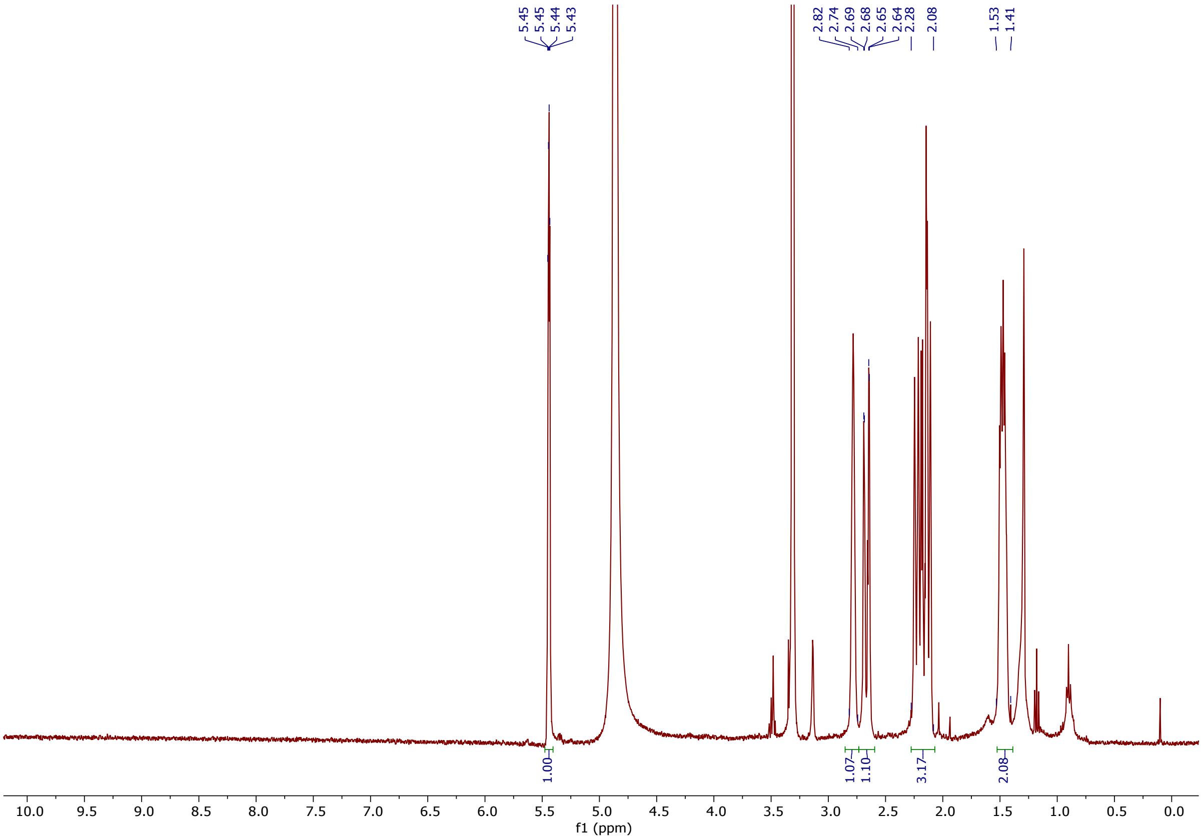

^{1}H NMR spectra of compound ***d$_5$*-1b** (400 MHz, 298K, $CDCl_3$)

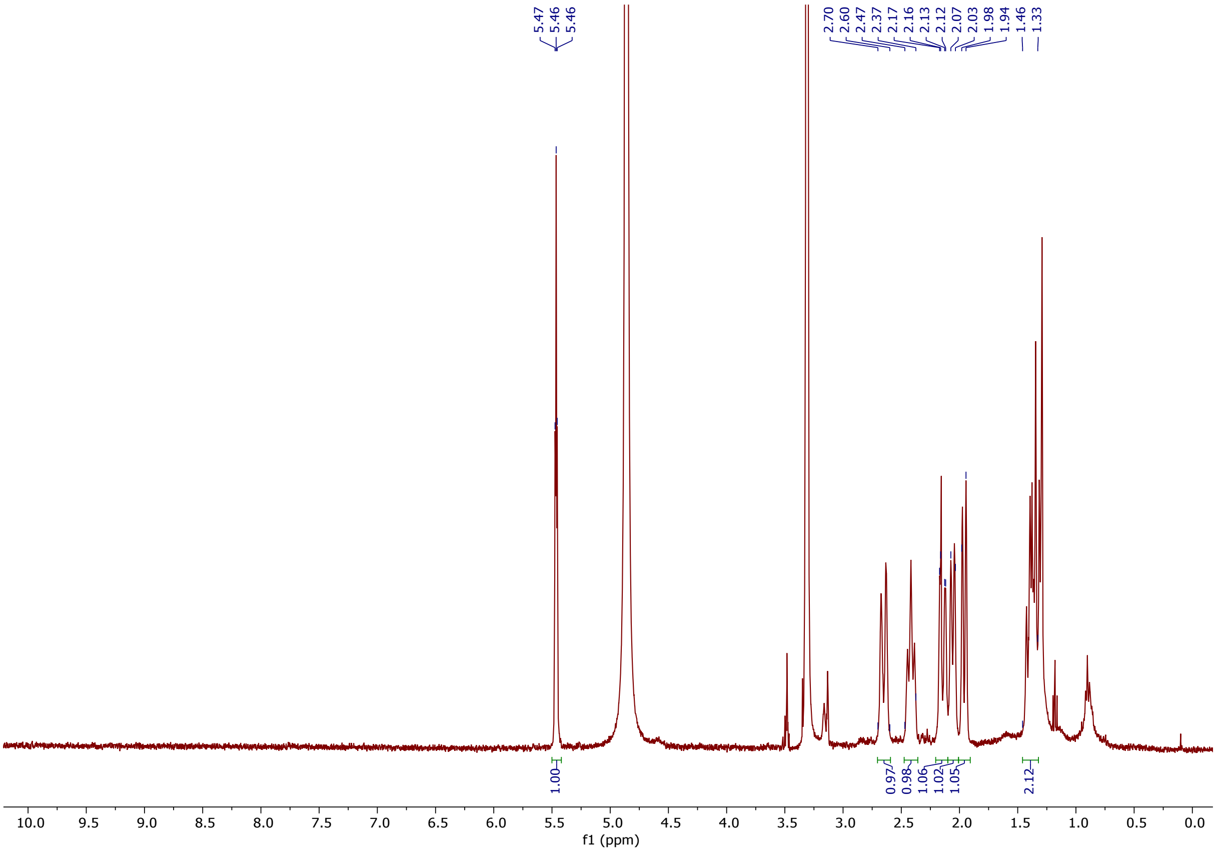

^{13}C NMR spectra of compound ***d$_5$*-1**, peaks picked for major isomer (126 MHz, 298K, $CDCl_3$)

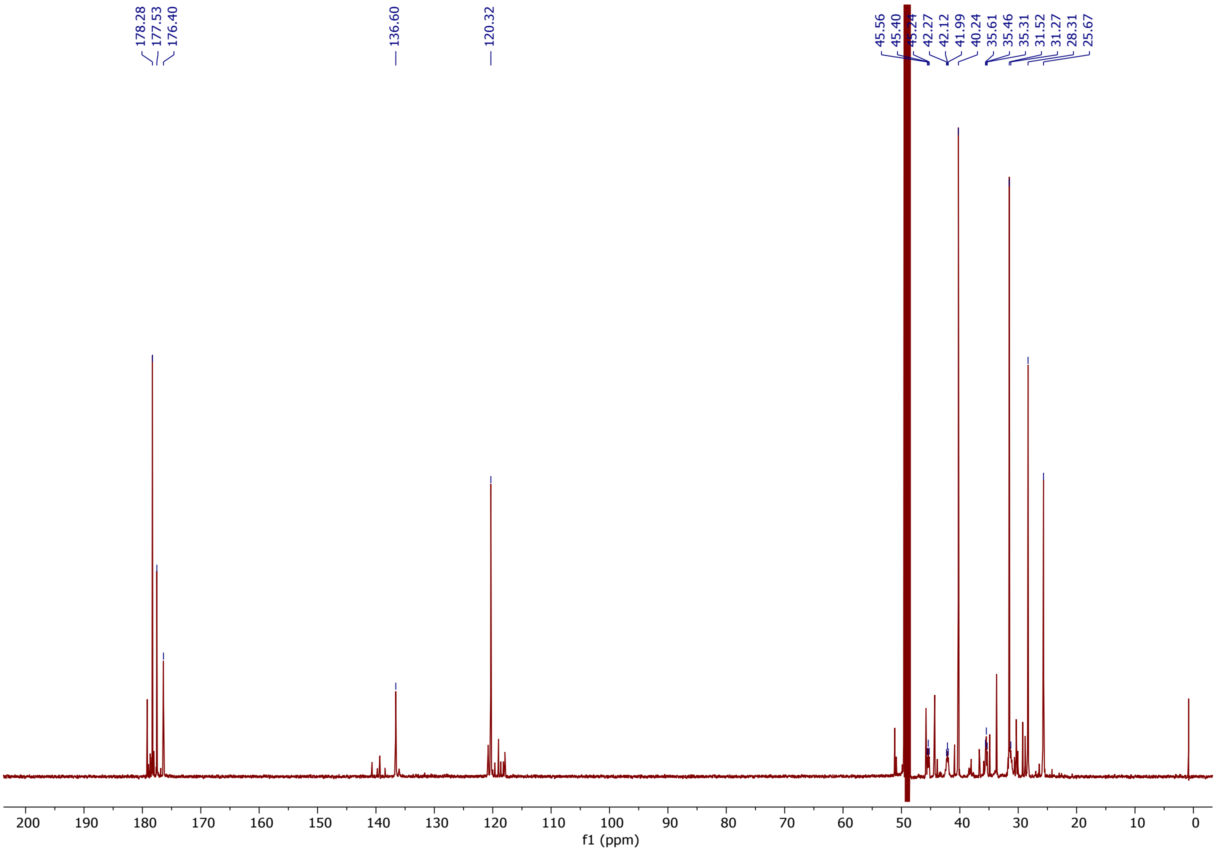

^{13}C NMR spectra of compound ***d$_5$*-1**, peaks picked above an arbitrary threshold (126 MHz, 298K, $CDCl_3$)

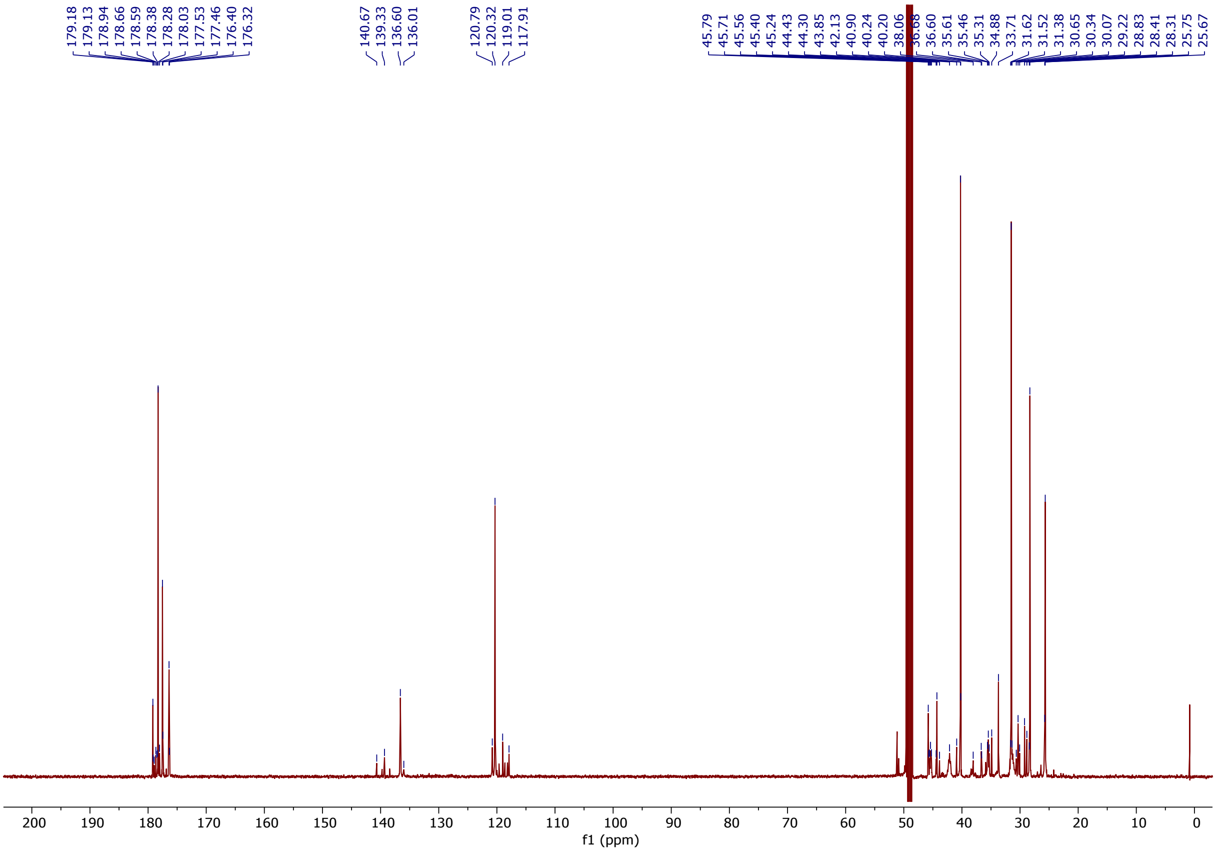

Bibliography

(1) Craig, A. J.; Moodie, L. W.; Hawkes, J. A. Preparation of Simple Bicyclic Carboxylate-Rich Alicyclic Molecules for the Investigation of Dissolved Organic Matter. *Environ. Sci. Technol.* **2024**, *58* (16), 7078–7086.
