# Supplementary figures and images for: Improvement of Electrospray Ionization Response Linearity and Quantification in Dissolved Organic Matter Using Synthetic Deuterated Internal Standards
Source: Anal Chem. 2025 Aug 22;97(34):18562–72. doi: 10.1021/acs.analchem.5c02463 (PMC12409698; doi:10.1021/acs.analchem.5c02463)

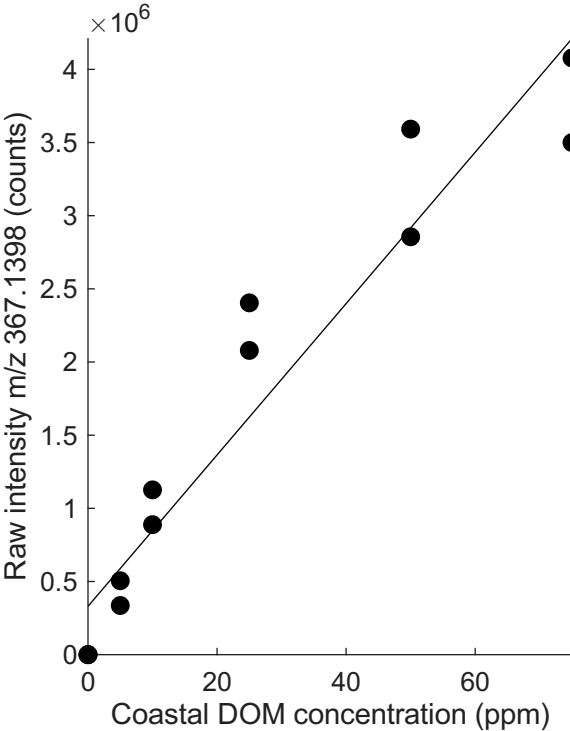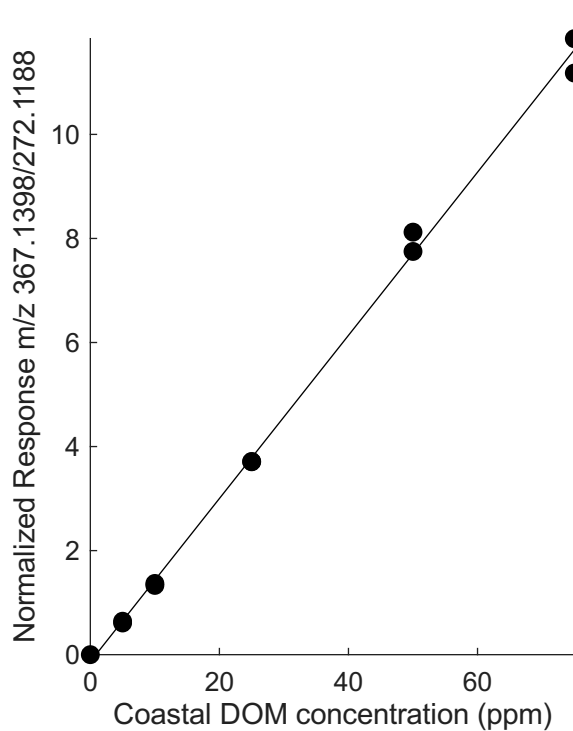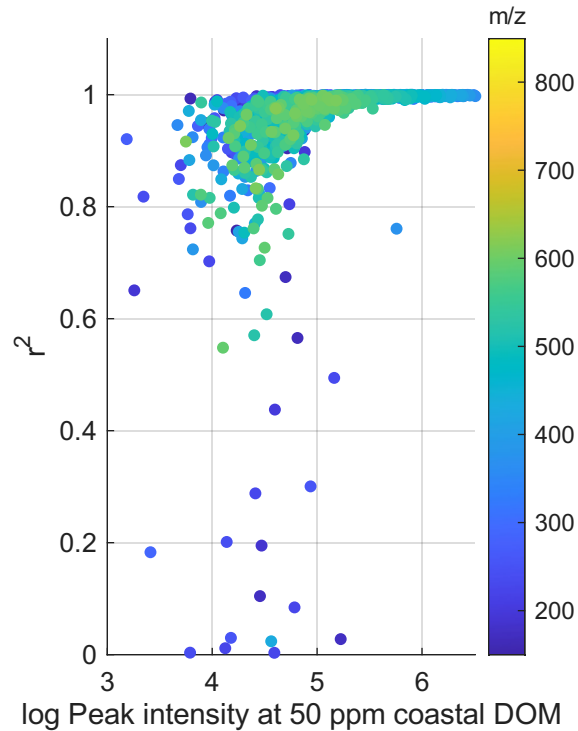

Supplement: Supplementary file 2 [file ac5c02463_si_002.zip › Final code for SI Aug15/TRM concentration series/Figure_2Matlab.pdf]
